# CARM1 and Paraspeckles Regulate Pre-implantation Mouse Embryo Development

**DOI:** 10.1016/j.cell.2018.11.027

**Published:** 2018-12-13

**Authors:** Anna Hupalowska, Agnieszka Jedrusik, Meng Zhu, Mark T. Bedford, David M. Glover, Magdalena Zernicka-Goetz

**Affiliations:** 1 Department of Physiology, Development and Neuroscience, University of Cambridge, Downing Street, Cambridge CB2 3EG, UK; 2 Department of Genetics, University of Cambridge, Downing Street, Cambridge CB2 3EG, UK; 3 Department of Epigenetics and Molecular Carcinogenesis, MD Anderson Cancer Center, The University of Texas, 1808 Park Road 1C, Smithville, TX 78957, USA

**Keywords:** paraspeckles, nuclear bodies, phase-separation, CARM1, p54/nrb, pre-implantation, embryo, development, heterogeneity, cell fate

## Abstract

Nuclear architecture has never been carefully examined during early mammalian development at the stages leading to establishment of the embryonic and extra-embryonic lineages. Heterogeneous activity of the methyltransferase CARM1 during these stages results in differential methylation of histone H3R26 to modulate establishment of these two lineages. Here we show that CARM1 accumulates in nuclear granules at the 2- to 4-cell stage transition in the mouse embryo, with the majority corresponding to paraspeckles. The paraspeckle component *Neat1* and its partner p54nrb are required for CARM1’s association with paraspeckles and for H3R26 methylation. Conversely, CARM1 also influences paraspeckle organization. Depletion of *Neat1* or p54nrb results in arrest at the 16- to 32-cell stage, with elevated expression of transcription factor Cdx2, promoting differentiation into the extra-embryonic lineage. This developmental arrest occurs at an earlier stage than following CARM1 depletion, indicating that paraspeckles act upstream of CARM1 but also have additional earlier roles in fate choice.

## Highlights

• CARM1 accumulates in paraspeckles in the 2- to 4-cell stage mouse embryo
• Paraspeckles correlate with heterogeneity of H3R26 methylation
• *Neat1* and p54nrb are required for CARM1’s association with paraspeckles
• Depletion of *Neat1* or p54nrb elevates differentiation marker Cdx2

## Graphical Abstract

**Figure:**
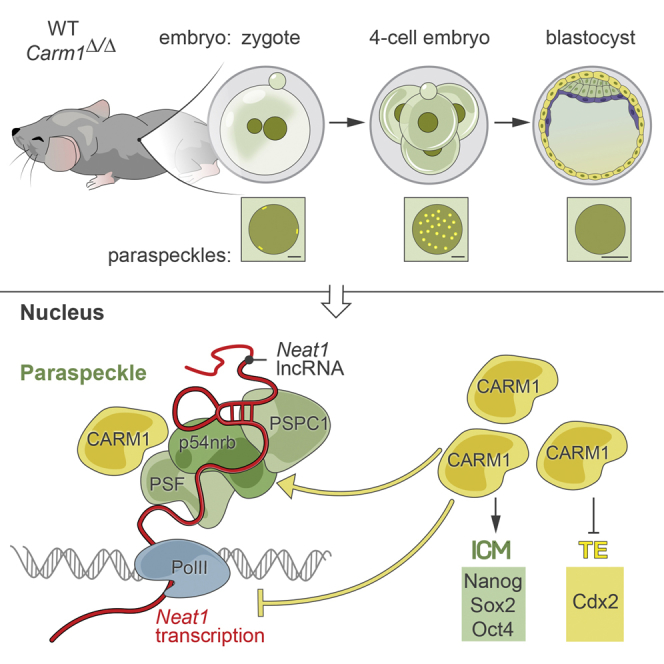

## Teaser

The association of the methyltransferase CARM1 with paraspeckles in 2- to 4-cell stage mouse embryos impacts proper lineage allocation and pre-implantation development.

## Introduction

**Figure 1:**
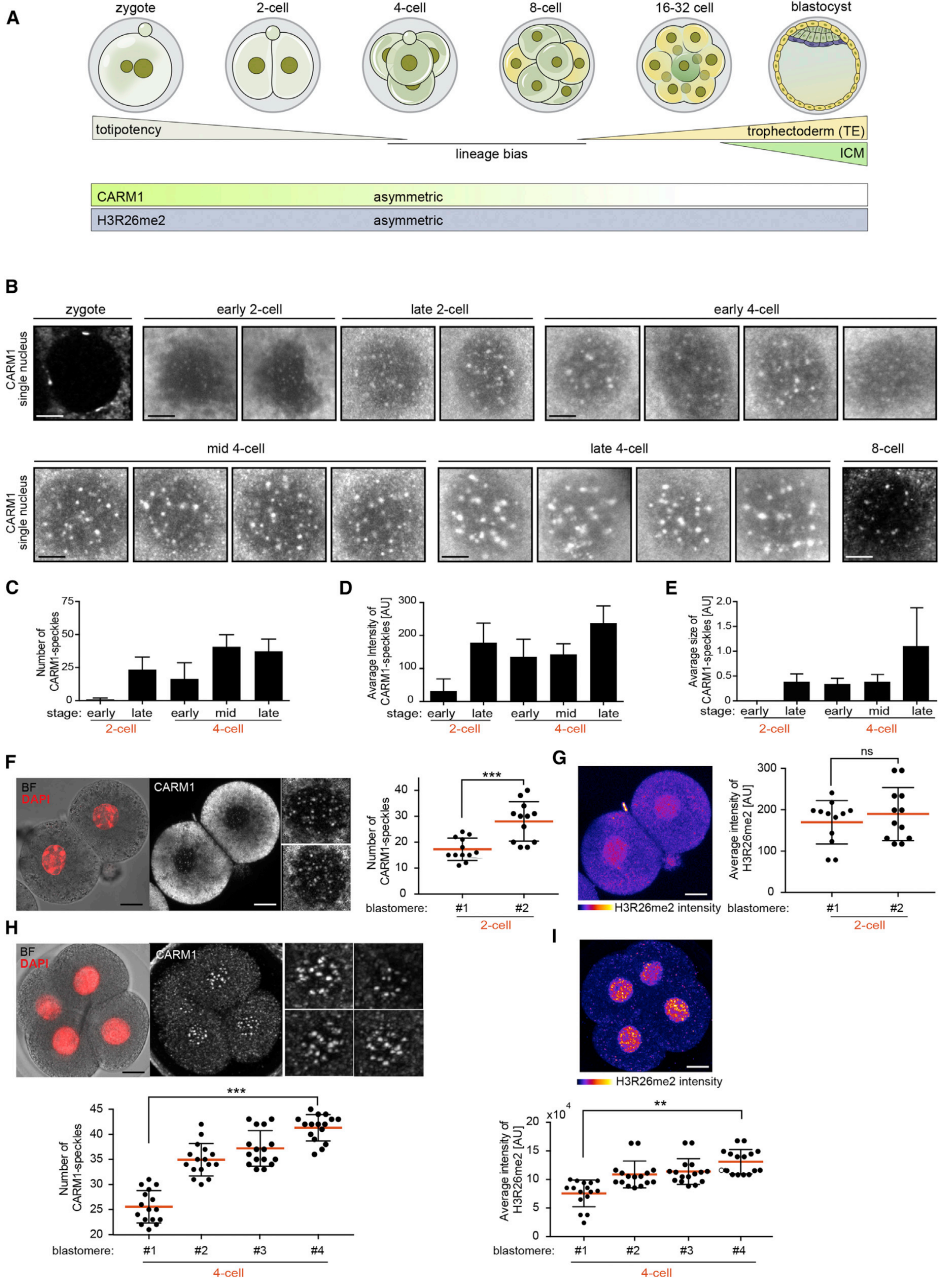
CARM1 Accumulates in Nuclear Granules at 2- and 4-Cell Stage Embryos (A) Stages of mouse embryo development between fertilization and implantation. The 8- to 16-cell division stage gives rise to inner (green) and outer (yellow) cells that contribute, respectively, to the inner cell mass (ICM) and trophectoderm (TE) of the blastocyst. CARM1 and H3R26me2 are asymmetrically distributed between cells at the 4-cell stage embryo. (B) CARM speckles in the individual nuclei from 2- and 4-cell embryos. Scale bars, 5 μm. (C–E) Quantification of the number (C), average intensity (D), and size (E) of CARM1-labeled speckles (n = 15 early 2-cell, n = 16 late 2-cell, n = 34 early 4-cell, n = 20 mid 4-cell, n = 32 late 4-cell embryos). (F) Differential numbers of CARM1 in 2-cell embryos (n = 12). Scale bars, 10 μm. Quantification, right; Mann-Whitney test, p = 0.0008. (G) Differential intensity of H3R26 staining in 2-cell embryos. Scale bars, 10 μm. Quantification, right; Mann-Whitney test, p = 0.5039. (H) Differential numbers of CARM1 in 4-cell embryos (n = 16). Scale bars, 10 μm. Quantification, right; ANOVA test, p < 0.0001. (I) Differential intensity of H3R26 immunofluorescence in 4-cell embryos. Scale bars, 10 μm. Quantification, right; ANOVA test, p < 0.0001. Error bars represent SEM.

Nuclear organization during pre-implantation development of the mouse embryo displays features necessary for the reprogramming of chromatin (Morgan et al., 2005). These involve histone modifications, nuclear repositioning, and the reorganization of chromatin associated with activation of specific genes. These changes occur in the embryo after fertilization and are necessary for establishment of the three lineages of the blastocyst: the pluripotent epiblast (EPI), which gives rise to the future body of the animal and that, together with extra-embryonic primitive endoderm (PE), is derived from the inner cell mass (ICM), and the trophectoderm (TE), the other extra-embryonic tissue that forms the placenta. It has been shown that differences in epigenetic modification between early blastomeres are linked to their fate. Therefore, cells with increased histone H3 arginine 26 methylation (H3R26me2), considered an activating mark, show higher expression of a subset of pluripotency genes that include *Nanog* and *Sox2* and are destined to contribute to embryonic rather than extra-embryonic tissues (Torres-Padilla et al., 2007, Goolam et al., 2016, White et al., 2016). Differential levels of histone H3R26me2 between 4-cell blastomeres are mediated by the heterogeneous activity of the histone coactivator associated arginine methyltransferase 1 (CARM1) (Torres-Padilla et al., 2007, Parfitt and Zernicka-Goetz, 2010, Shi et al., 2015; Figure 1A). However, nuclear organization and its potential effect on gene expression and, specifically, lineage allocation during pre-implantation development have not been addressed extensively and await further investigation.

The nuclei of higher eukaryotes contain multiple nuclear bodies that mediate distinct molecular processes, ranging from DNA replication to RNA transcription and processing. Studies of the dynamics of nuclear structures in the mammalian embryo have predominantly focused on nucleoli and Cajal bodies (Ferreira and Carmo-Fonseca, 1995, Fléchon and Kopecný, 1998, Zatsepina et al., 2003). Other nuclear domains, such as interchromatin granule clusters (IGCs), perichromatin granules (PGs), nuclear speckles, and paraspeckles and their related proteins, have so far not been studied in detail or not at all in the mammalian embryo.

Paraspeckles are observed within IGCs and were initially defined as foci enriched in characteristic RNA-binding proteins, including the three mammalian DBHSs (*Drosophila* behavior and human splicing) proteins: PSPC1, p54nrb (NonO), and SFPQ (PSF) (Fox et al., 2002, Prasanth et al., 2005). These are membrane-less, dynamic structures working as open systems as their components exchange with freely diffusing molecules in the nucleoplasm (Mao et al., 2011).

Paraspeckles are built around scaffolds of a specific long noncoding RNA (lncRNA) known as nuclear paraspeckle assembly transcript 1 (*Neat1*). *Neat1* and its ongoing transcription are required for the structural integrity of paraspeckles (Sasaki et al., 2009, Sunwoo et al., 2009, Mao et al., 2011). It has been reported that paraspeckles respond dynamically to a variety of basic physiological processes such as cell differentiation, viral infection, altered metabolic conditions, and signaling (Clemson et al., 2009, Hutchinson et al., 2007, Sone et al., 2007, Sasaki et al., 2009, Sunwoo et al., 2009, Zheng et al., 2010, Yang et al., 2011). Paraspeckles enable nuclear retention of certain mRNAs, decreasing their translation (Anantharaman et al., 2016). They also sequester certain RNA binding proteins (RBPs) to limit their functions in the nucleus (Hu et al., 2015, Prasanth et al., 2005, Chen and Carmichael, 2009, Mao et al., 2011, Chen et al., 2008).

It has been demonstrated that CARM1 interacts with paraspeckles through p54nrb (Hu et al., 2015). Although it is known that CARM1 is associated with transcriptional activation and that its differential activity between blastomeres has an effect on lineage allocation, its exact mechanism of action needs further investigation. Here we wished to test the hypothesis that nuclear organization of blastomeres has an effect on proper lineage allocation and pre-implantation development and that this process involves CARM1.

## Results

### CARM1 Speckles Appear Heterogeneously at the 2- to 4-Cell Stage Transition

**Figure S1:**
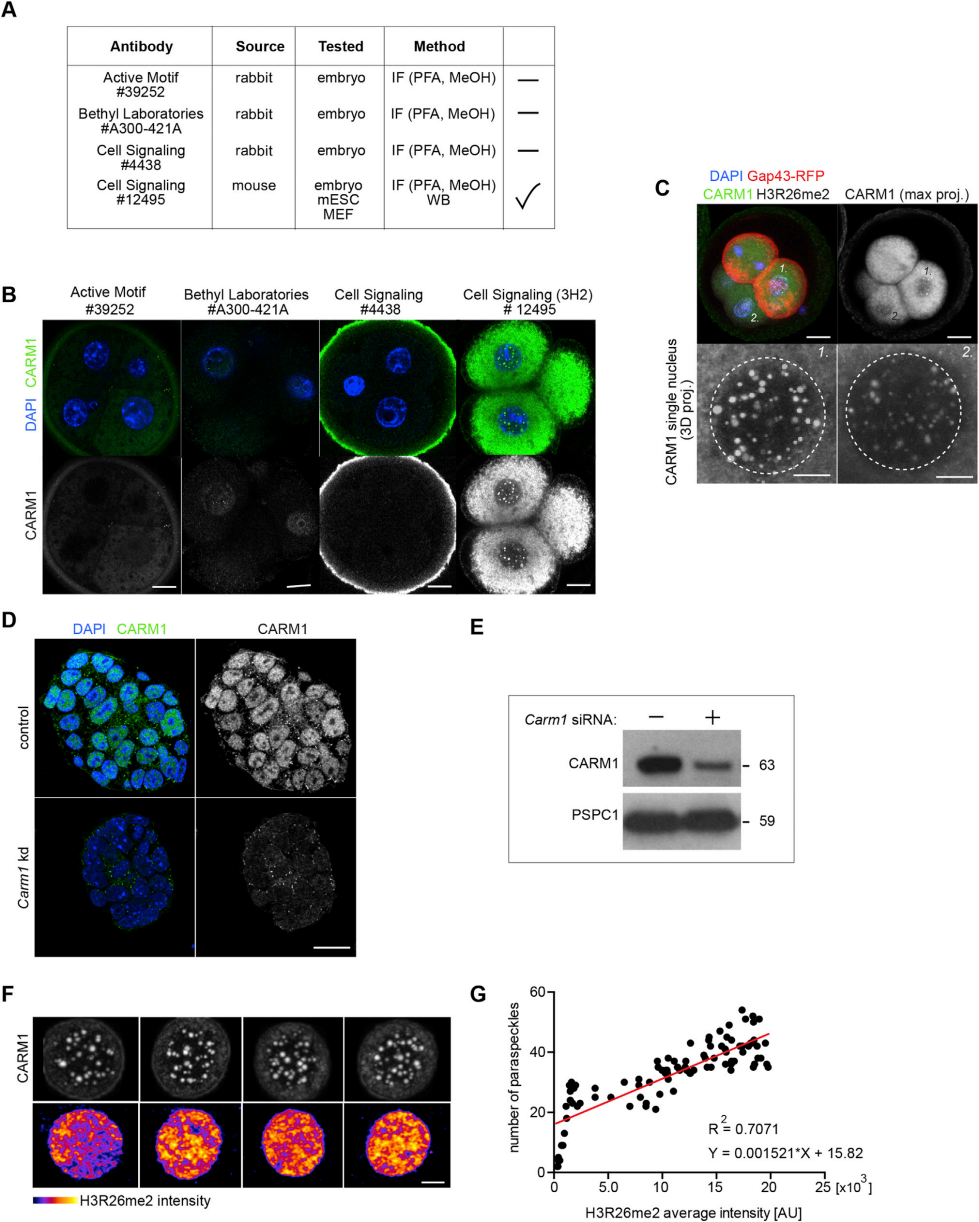
CARM1 Accumulates in the Nucleus of Mouse Embryos and mESCs; Validation of Antibodies, Related to Figure 1 (A) Assessment of anti-CARM1 antibodies in immunofluorescense and western blots. (B) Staining to reveal CARM1 and DNA (DAPI) in the 4-cell mouse embryo with different anti-CARM1 antibodies. Scale bar 10 μm. (C) One blastomere of 2-cell stage embryo was injected with *Carm1*-WT synthetic mRNA and *Gap43*-RFP mRNA. Embryos were cultured until the 4-cell stage and subjected to immunostaining for CARM1 (#12495). (D) Confocal images of CARM1 in mESCs transfected with either control or *Carm1* siRNAs. Scale bar 20 μm. (E) *Carm1* knockdown reduces level of CARM1 protein in ESCs. mESCs were transfected either with control or *Carm1* siRNAs for 48 h. Cell lysates were analyzed by western blotting for CARM1 and PSPC1 (loading control). (F) Differential numbers of CARM1-speckles and intensity of H3R26 immunofluorescence in single nuclei of 4-cell embryos. Nuclei of a representative embryo are shown. Scale bar 10 μm. (G) Spearman correlation coefficients of the expression of H3R26me2 and the number of CARM1 speckles (n = 99 nuclei, p < 0.0001).

Histone H3R26 methylation mediated by CARM1 has been reported to be heterogeneously distributed between blastomeres of 4-cell stage mouse embryos (Torres-Padilla et al., 2007), but the nuclear distribution of CARM1 remained unknown. To study CARM1’s nuclear distribution, we first selected an antibody with high specificity against CARM1 in immunofluorescence and western blots (Figures S1A–S1E). Using this specific anti-CARM1 antibody, we identified numerous bright foci of CARM1 staining appearing in the nucleoplasm of 2- and 4-cell stage embryos that became weaker and diffuse in the nucleoplasm by the late 8-cell stage (Figure 1B).

Because CARM1 speckles showed a marked increase in number (Figure 1C), intensity (Figure 1D), and size (Figure 1E), specifically in the 2- and 4-cell stage embryo, we focused on analyzing their distribution at these stages. We found that, at the 2-cell stage, the number of CARM1 speckles differed between sister blastomeres, with one cell having an average of 16 such speckles (17.25 ± 1.244, n = 12) and the other 28 (28.00 ± 2.198, n = 12). This difference did not correlate with any changes in histone H3R26me2 staining between blastomeres at this stage (Figures 1F and 1G). Similarly, the number of CARM1 speckles differed between cells in the 4-cell embryo, with one blastomere having a significantly lower number of CARM1 speckles (26 on average; 26.30 ± 1.55, n = 16) in comparison with others (40 on average; 40.30 ± 1.713, n = 16) (Figures 1H and 1I). This difference directly correlated with differential levels of H3R26me2 and was maximal in blastomeres with the highest level of H3R26me2 (Figure 1I; Figures S1F and S1G), shown previously to bias blastomeres to an embryonic rather than extra-embryonic fate. These results indicate that heterogeneity of the numbers of CARM1 speckles and the extent of the histone H3R26me2 modification correlate at the 4-cell stage.

### CARM1 Accumulates in Nuclear Paraspeckles

**Figure 2:**
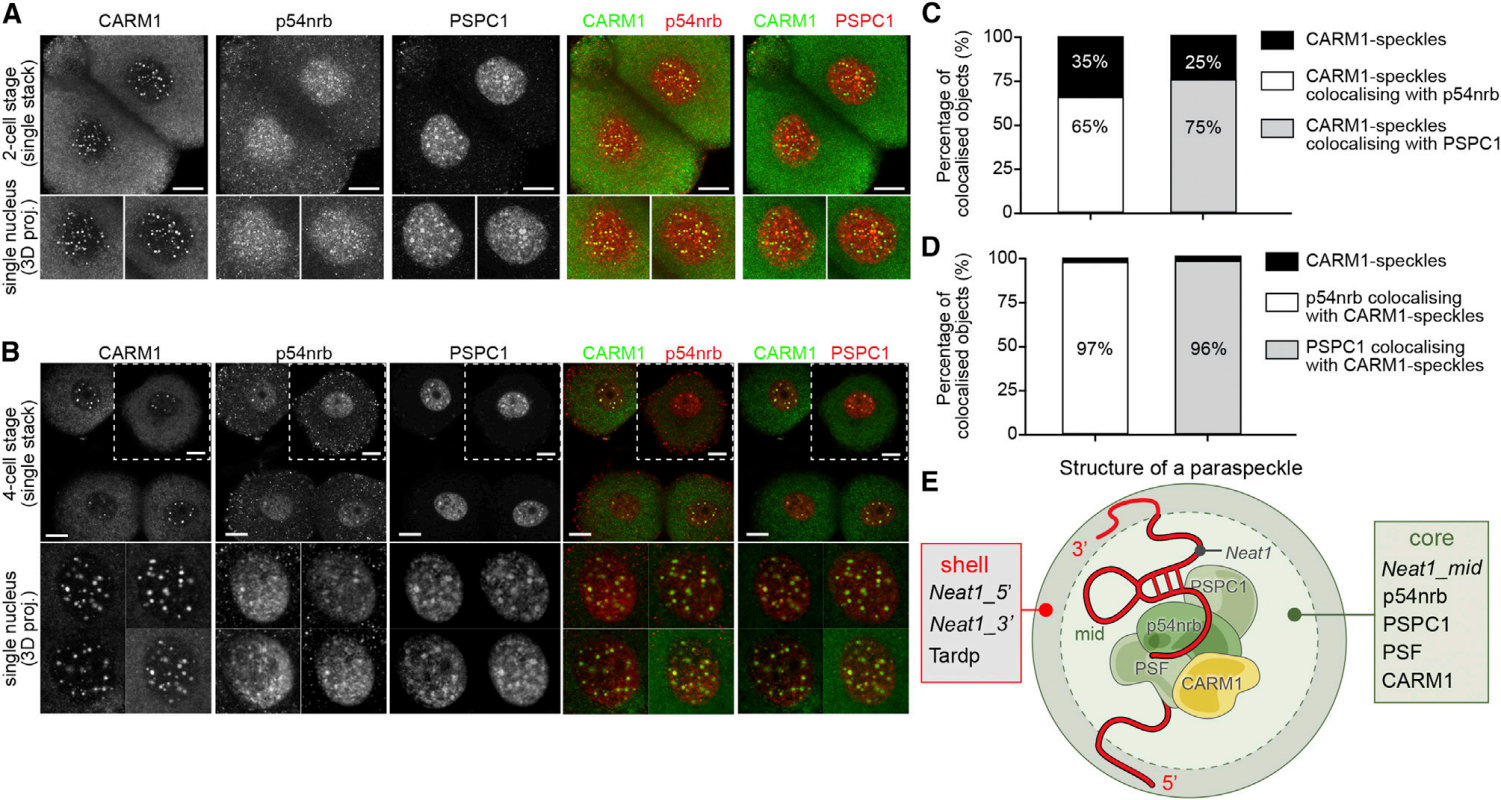
CARM1 Accumulates in Nuclear Paraspeckles (A and B) Co-immunostaining of CARM1 with the paraspeckle components p54nrb and PSPC1 at the 2-cell stage (A) and 4-cell stage (B). A magnified view of single nuclei is presented at the bottom. Scale bars, 10 μm. (C) Quantification of CARM1-positive structures co-staining with p54nrb and PSPC1 (20 × 4-cell embryos, 80 nuclei in total). (D) Quantification of p54nrb and PSPC1 co-staining with CARM1-positive structures in the nucleus (20 × 4-cell embryos, 80 nuclei in total). (E) Graphical representation of a paraspeckle.

**Figure S2:**
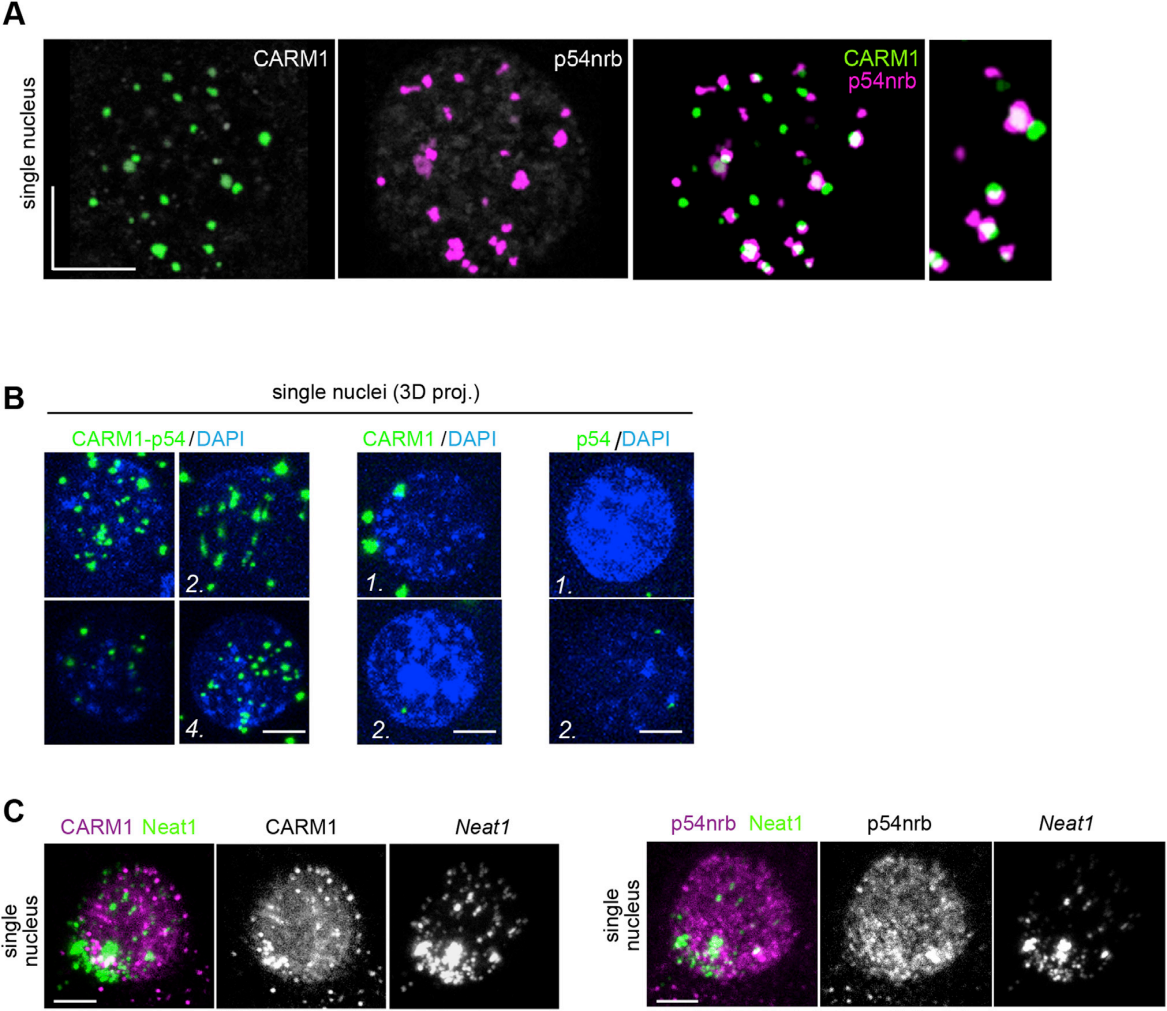
CARM1 Colocalizes with Paraspeckle Components, Related to Figure 2 (A) Segmentation of a single nucleus from the 4-cell stage embryo. Co-immunostaining of CARM1 with paraspeckle component, p54nrb (20 × 4-cell embryos, 80 nuclei in total). Scale bar 10 μm. (B) Proximity of CARM1 and p54nrb analyzed at the 4-cell stage by proximity ligation assay (10 × 4-cell embryos, 40 nuclei in total). Scale bar 10 μm. (C) Immuno-localization of CARM1 and p54nrb with *Neat1* RNA detected by *in situ* hybridization (10 × 4-cell embryos, 40 nuclei in total). Scale bar 5 μm. Error bars represent s.e.m.

Our above observations suggested that the pattern of CARM1 staining resembles “nuclear speckles” or IGCs (Mintz et al., 1999). Among the IGCs are paraspeckles that characteristically contain a core component, p54nrb, that is also a substrate for CARM1 (Hu et al., 2015). To assess whether CARM1 colocalizes with paraspeckles in the mouse embryo, we next co-stained embryos to reveal CARM1 and two core paraspeckle components, p54nrb and PSPC1. We found an extensive overlap of the staining pattern of CARM1 with either p54nrb or PSPC1 at the 2- and 4-cell stages (Figures 2A, 2B, and S2A), suggesting that the majority of CARM1 was associated with paraspeckles. However, a significant proportion of CARM1 was also present in other nuclear speckles. Specifically, 65% ± 7.5% of CARM1 speckles (mean ± SD) co-stained for p54nrb, and 75% ± 9.7% of CARM1 speckles (mean ± SD) were PSPC1-positive (Figure 2C). The presence of CARM1 in the close vicinity of paraspeckle components at the 4-cell stage was also suggested by proximity ligation assays (Figure S2B) and by its immuno-localization at the site of *Neat1* RNA detected by *in situ* hybridization (Figure S2C). Although approximately one-third of CARM1 was present in bodies not associated with paraspeckle markers, 97% ± 1% of p54nrb and 96% ± 2% of PSPC1 speckles co-stained for CARM1 (Figure 2D). These results indicate that, effectively, all paraspeckle structures in the 4-cell stage mouse embryo contain CARM1 and that these represent about two-thirds of the CARM1 speckles (Figure 2E). They also indicate that CARM1 speckles may represent distinct subpopulations of nuclear bodies in the embryo, with the major proportion associated with paraspeckles.

### A Link between Paraspeckle Components and CARM1 Levels

**Figure 3:**
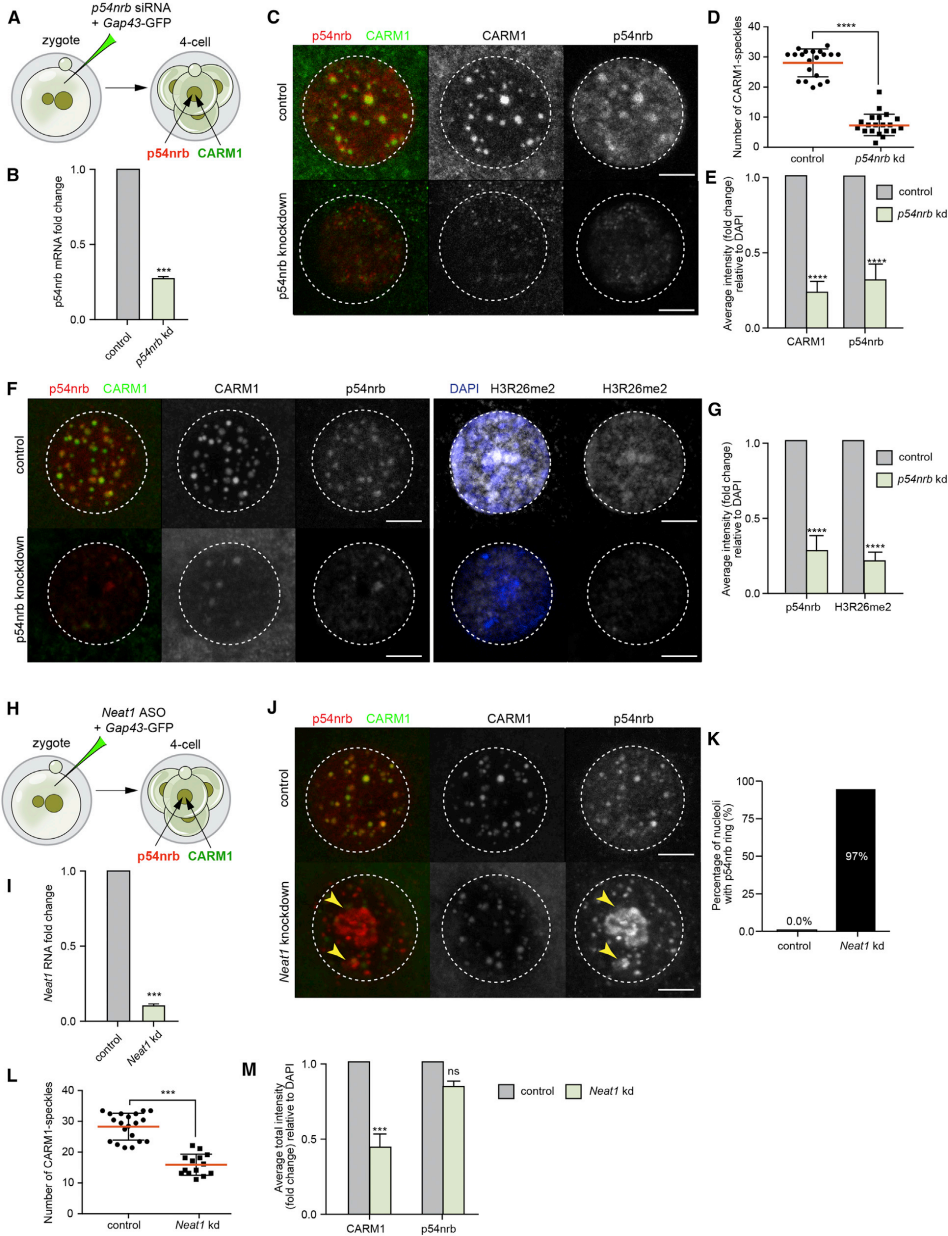
CARM1 Levels Depend on Paraspeckle Components (A) Scheme of the *p54nrb* knockdown experiment. Zygotes were injected with *p54nrb* siRNA or control siRNA along with the *Gap43*-GFP mRNA (injection control). Embryos were cultured until the 4-cell stage and either subjected to RNA isolation and qRT-PCR or immunostaining for the indicated markers. (B) qRT-PCR of 4-cell stage embryos injected with either control or *p54nrb* siRNA (n = 90, 3 biological replicates), validating *p54nrb* knockdown efficiency (Student’s t test, p < 0.001). (C) Confocal images of representative embryos showing CARM1 and p54nrb localization in control and *p54nrb*-depleted embryos in a representative individual nucleus from a 4-cell stage embryo. Scale bars, 5 μm. (D) Quantification of the number of CARM1 speckles in control and *p54nrb*-depleted 4-cell stage embryos (20 × 4-cell embryos, 80 nuclei in total; Mann-Whitney test, p < 0.0001). (E) Quantification of the relative fluorescence intensity of CARM1 and p54nrb in control and *p54nrb*-depleted 4-cell stage embryos (20 × 4-cell embryos, 80 nuclei in total; Mann-Whitney test, p < 0.0001). (F) Confocal images showing CARM1, p54nrb, and H3R26me2 localization in control and *p54nrb*-depleted embryos in a representative nucleus from a 4-cell stage embryo (20 × 4-cell embryos, 80 nuclei in total). Scale bars, 5 μm. (G) Quantification of the relative fluorescence intensity of p54nrb and H3R26me2 in control and *p54nrb*-depleted 4-cell stage embryos (20 × 4-cell embryos, 80 nuclei in total; Mann-Whitney test, p < 0.0001). (H) Schematic showing *Neat1* depletion with antisense oligonucleotides (ASOs). Zygotes were injected with *Neat1* or control ASOs along with the *Gap43*-GFP mRNA (injection control). Embryos were cultured until the 4-cell stage and either subjected to RNA isolation and qRT-PCR or immunostaining for the indicated markers. (I) qRT-PCR of 4-cell stage embryos injected with either control or *Neat1* ASOs (n = 75, 3 biological replicates), validating *Neat1* knockdown efficiency (Student’s t test, p < 0.001). (J) Confocal images of CARM1 and p54nrb localization in a representative nucleus of a control and *Neat1* depleted 4-cell stage embryo. Yellow arrowheads indicate p54nrb clusters. Scale bars, 5 μm. (K) Quantification of p54nrb structures localized around the nucleoli in control and *Neat1*-depleted 4-cell stage embryos (46 × 4-cell embryos, 184 nuclei in total). (L) Quantification of a number of CARM1 speckles in control and *Neat1*-depleted 4-cell stage embryos (46 × 4-cell embryos, 184 nuclei in total; Mann-Whitney test, p < 0.0001). (M) Quantification of the relative fluorescence intensity of CARM1 and p54nrb in control and *Neat1*-depleted 4-cell stage embryos (46 × 4-cell embryos, 184 nuclei in total; Mann-Whitney test, p < 0.0001). Error bars represent SEM.

**Figure S3:**
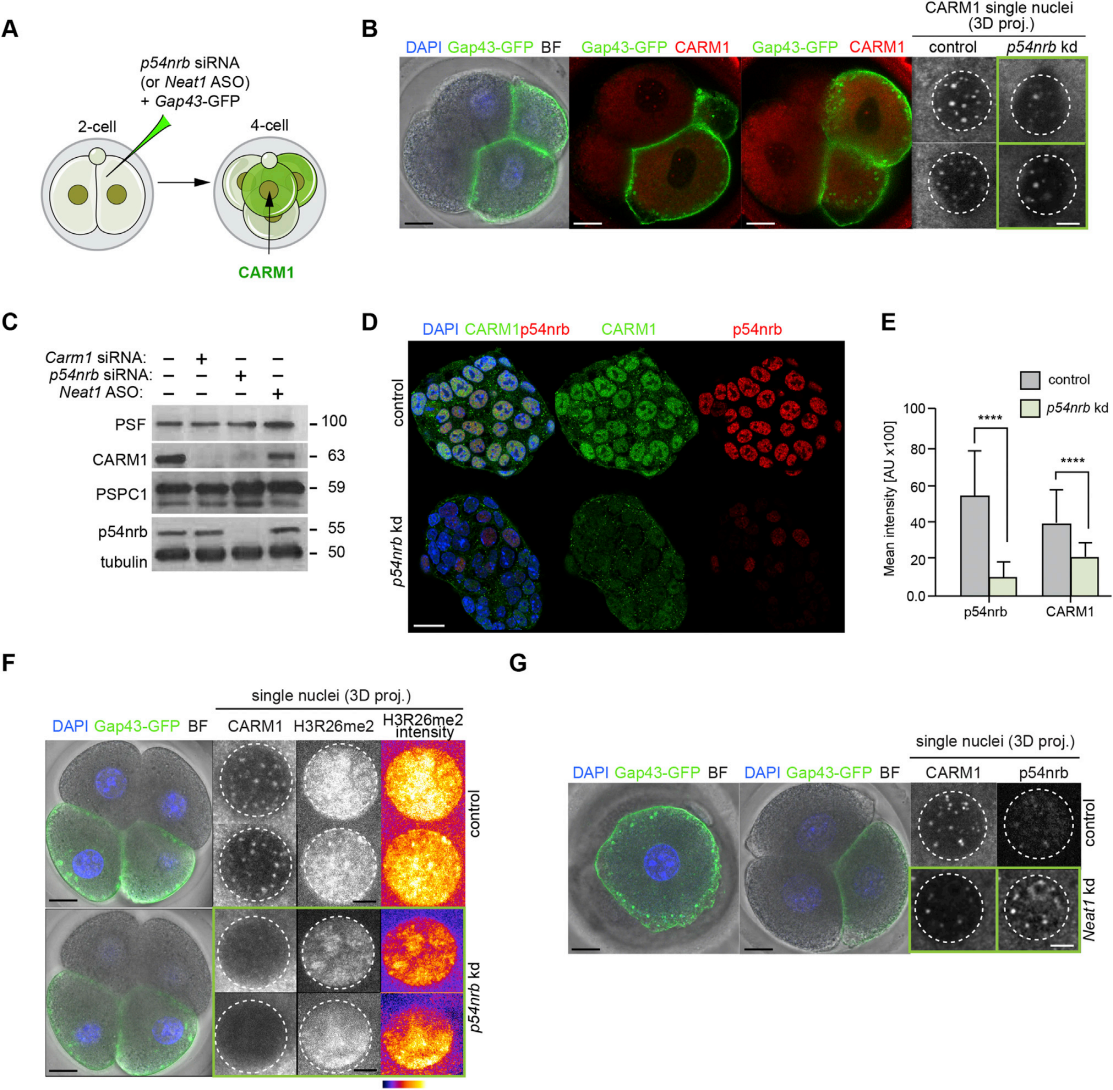
CARM1 Levels Depend on *p54nrb* and *Neat1*, Related to Figure 3 (A) Scheme of *p54nrb* knockdown experiment. One blastomere of a 2-cell stage embryo was injected with *p54nrb* siRNA or control siRNA together with the *Gap43*-GFP synthetic mRNA (injection control). Embryos were cultured until the 4-cell stage and subjected to immunostaining for the indicated markers. (B) Confocal images of CARM1 and p54nrb localization in control and p54nrb depleted blastomeres (n = 18). Nuclei of a 4-cell stage embryo are shown. Scale bar 5mm. (C) *p54nrb* knockdown reduces levels of CARM1 protein in ESCs. mESCs were transfected with control, *Carm1*, or *p54nrb* siRNAs or with *Neat1* ASO for 48 h. Cell lysates were analyzed by western blotting for PSF, CARM1, PSPC1, p54nrb and tubulin (loading control). (D) Confocal images of CARM1 and p54nrb in mESCs transfected with either control or *p54nrb* siRNAs. Scale bar 20 μm. (E) Quantification of relative fluorescence intensity of CARM1 and p54nrb in control and p54nrb-depleted mESCs (Mann-Whitney test, p < 0.0001). (F) Reduction in H3R26me2 levels in progeny of single cell injected with *p54nrb* siRNA at 2-cell stage (n = 10). Scale bar 5 μm. (G) Reduction in CARM1 and redistribution of p54nrb in progeny of single cell injected with *Neat1* ASOs at 2-cell stage (n = 10). Scale bar 5 μm. Error bars represent s.e.m.

Because our results revealed that the majority of CARM1 is associated with paraspeckles, we next wished to determine the consequences of disrupting paraspeckle function by depleting their essential components in the early embryo. We first depleted p54nrb in the zygote by injecting small interfering RNA (siRNA) targeting *p54nrb*, together with synthetic mRNA for membrane-bound *Gap43*-GFP to mark the injected cells and examined the distribution of CARM1-labeled speckles at the 4-cell stage (Figure 3A). The efficiency of *p54nrb* knockdown was confirmed by RT-PCR (Figure 3B). We found that both the number of CARM1 speckles and CARM1 intensity were reduced upon depletion of p54nrb (Figures 3C–3E). Detailed analysis showed that, in p54nrb-depleted embryos, CARM1-positive structures were reduced by 2.7-fold and the average intensity of CARM1 by 4.0-fold (Figures 3D and 3E). This observation was confirmed when p54nrb was depleted in a single blastomere of the 2-cell stage embryo (Figures S3A and S3B). These results are in accord with the finding that depletion of p54nrb from embryonic stem cells (ESCs) also resulted in loss of both p54nrb and CARM1 (Figures S3C–S3E). A residual pool of CARM1 containing nuclear structures, not affected by 54nrb depletion, could possibly represent the 35% of CARM1 structures not associated with paraspeckles.

The above findings raised the question of whether p54nrb might act in partnership with CARM1 to regulate CARM1 expression and/or activity. Because one function of CARM1 is to methylate histone H3 at R26, we asked whether depletion of p54nrb from the zygote stage onward would also reduce H3R26me2 levels. Indeed, we found that downregulation of p54nrb led to a reduction of CARM1 speckles and a reduction in H3R26me2 at distinct nuclear sites (Figures 3F and 3G). This observation was confirmed when p54nrb was downregulated in one of the 2-cell stage blastomeres, allowing us to compare CARM1 speckles and H3R26me2 in the treated and control halves of the embryo (Figures S3A and S3F). Thus, the association of CARM1 with paraspeckles reflects its ability to methylate H3R26 in the 4-cell stage embryo.

Paraspeckle integrity also requires ongoing transcription of *Neat1*, suggesting that paraspeckles are linked to *Neat1* biogenesis (Naganuma et al., 2012, Sasaki and Hirose, 2009, Sasaki et al., 2009). We argued, therefore, that if we were to deplete *Neat1*, then this might have a similar outcome as depleting p54nrb. To address this possibility, we injected zygotes with anti-sense oligonucleotides (ASOs) against *Neat1* and examined the structure of paraspeckles by analyzing their components, p54nrb and CARM1, at the 4-cell stage (Figure 3H). The efficiency of *Neat1* knockdown was confirmed with RT-PCR (Figure 3I). We observed that, in the majority of nuclei (97% ± 1.2%, mean ± SD), paraspeckles were disrupted, as judged by the re-localization of p54nrb from paraspeckles to the periphery of the nucleoli (Figures 3J and 3K). Both the number and the average intensity of CARM1 speckles were reduced by 2.0-fold (Figures 3L and 3M). To verify this result, we injected a single blastomere of 2-cell stage embryos with *Neat1* ASOs or control ASOs and cultured embryos until the 4-cell stage. We found that *Neat1* depletion resulted in re-localization of p54nrb from paraspeckles around the nucleoli and a reduced number of CARM1 speckles compared with control blastomeres (Figures S3A and S3G). Together, these results suggest that the functional ability of CARM1 to modify H3R26me2 requires paraspeckle integrity, itself dependent on *Neat1* RNA and p54nrb.

### A Link between CARM1 and Paraspeckle Behavior

**Figure 4:**
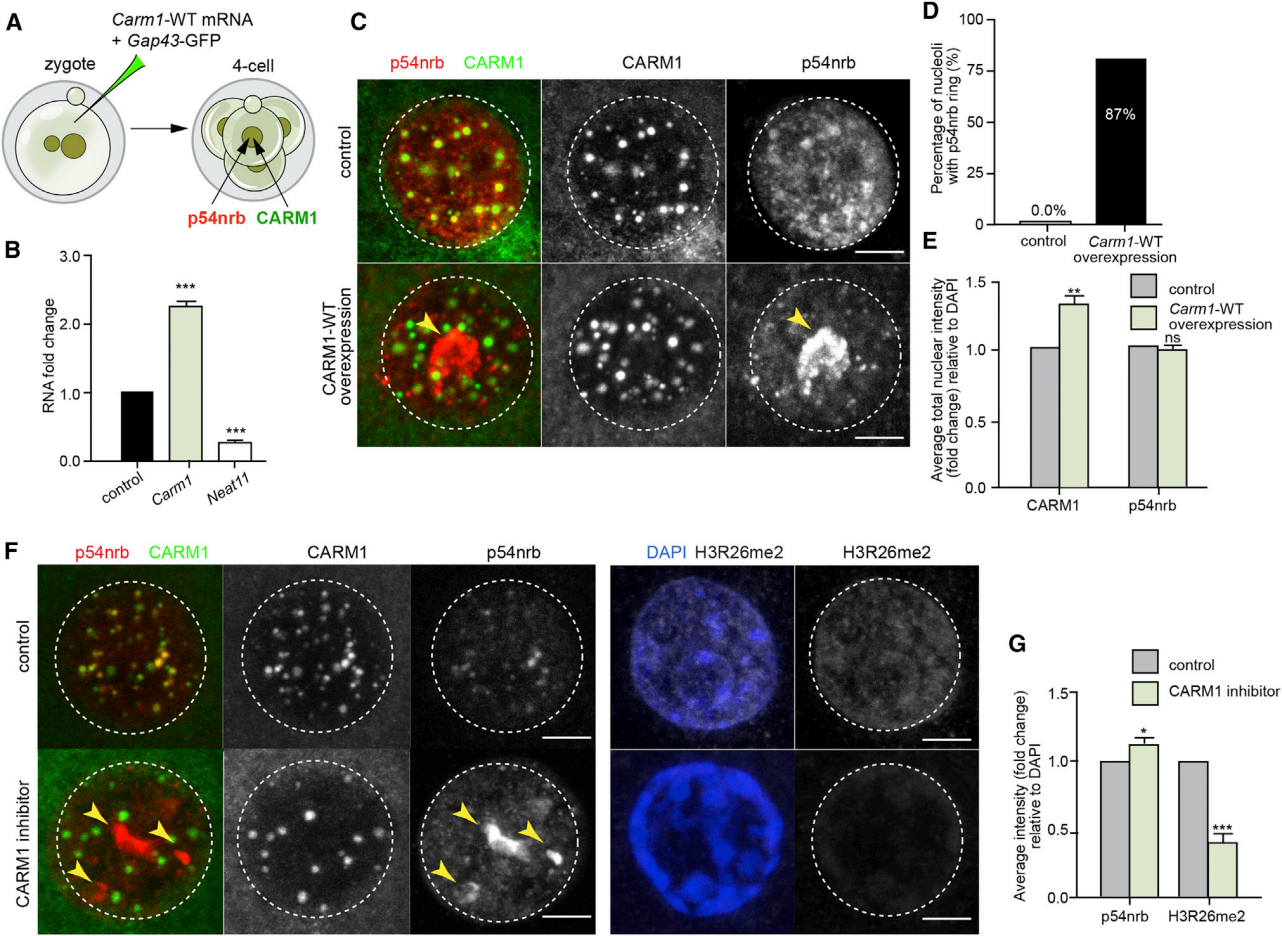
CARM1 Levels Modulate Paraspeckle Assembly (A) Scheme for overexpression of wild-type (WT) *Carm1* synthetic mRNA. Zygotes were injected with either *Carm1*-WT synthetic mRNA and *Gap43*-GFP mRNA or *Gap43*-GFP mRNA only as a control. Embryos were cultured until the 4-cell stage and either subjected to RNA isolation and qRT-PCR or immunostaining for the indicated markers. (B) qRT-PCR of 4-cell stage embryos injected with either control or *Carm1*-WT synthetic mRNA (n = 60, 2 biological replicates), validating *Carm1* mRNA and *Neat1* levels (Student’s t test, p < 0.001). (C) Confocal images of CARM1 and p54nrb localization in control embryos and with elevated levels of CARM1, showing a representative nucleus of a 4-cell stage embryo. Yellow arrowheads indicate p54nrb clusters. Scale bars, 5 μm. (D) Quantification of p54nrb structures localized around the nucleoli in control and CARM1-WT overexpressing 4-cell stage embryos (30 × 4-cell embryos, 120 nuclei in total). (E) Quantification of the relative fluorescence intensity of CARM1 and p54nrb in control and CARM1-WT overexpressing 4-cell stage embryos (30 × 4-cell embryos, 120 nuclei in total; Mann-Whitney test, p = 0.6829). (F) Confocal images of CARM1, p54nrb, and H3R26me2 localization in control embryos and with inhibited activity of CARM1, showing a representative nucleus of a 4-cell stage embryo. Yellow arrowheads indicate p54nrb clusters. Scale bars, 5 μm. (G) Quantification of the relative fluorescence intensity of p54nrb and H3R26me in control and 4-cell stage embryos treated with CARM1 inhibitor (34 × 4-cell embryos, 136 nuclei in total; Mann-Whitney test, p < 0.0001). Error bars represent SEM.

**Figure S4:**
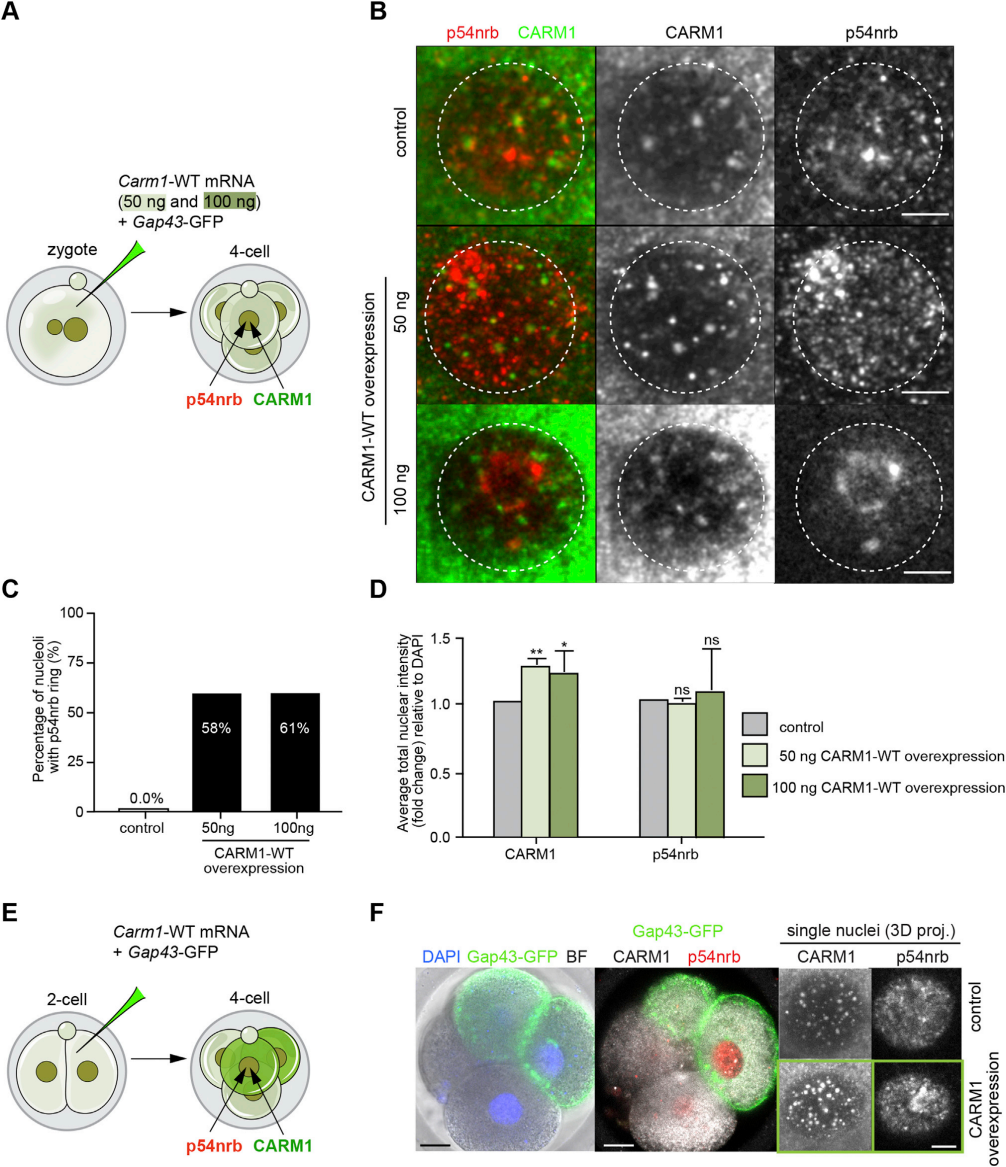
CARM1 Levels Modulate Paraspeckle Body, Related to Figure 4 (A) Scheme for overexpression of wild-type (WT) *Carm1* synthetic mRNA. Zygotes were injected with either *Carm1*-WT synthetic mRNA (50 ng or 100 ng) and *Gap43*-GFP mRNA only as control. Embryos were cultured until the 4-cell stage and subjected to immunostaining for the indicated markers. (B) Confocal images of CARM1 and p54nrb localization in control embryos and with elevated levels of CARM1 showing a representative nucleus of a 4-cell stage embryo. Yellow arrowheads indicate p54nrb clusters. Scale bar 5 μm. (C) Quantification of p54nrb structures localized around the nucleoli in control and CARM1-WT overexpressing 4-cell stage embryos (10 × 4-cell embryos, 40 nuclei in total per each condition). (D) Quantification of relative fluorescence intensity of CARM1 and p54nrb in control and CARM1-WT overexpressing 4-cell stage embryos (10 × 4-cell embryos, 40 nuclei in total per each condition; Mann-Whitney test, p = 0.5925 and p = 0.7042). (E) Scheme for overexpression of wild-type (WT) *Carm1* synthetic mRNA and *Gap43*-GFP mRNA in one blastomere of 2-cell stage embryo. Embryos were cultured until the 4-cell stage and subjected to immunostaining for the indicated markers. (F) Confocal images of CARM1 and p54nrb localization in control blastomeres and blastomeres with elevated levels of CARM1 (n = 20). Nuclei of a 4-cell stage embryo are shown. Note clustering of p54nrb in lineage of treated cell. Scale bar 5 μm. Error bars represent s.e.m.

CARM1 is enriched at the *Neat1* promoter to inhibit *Neat1* transcription, and its deficiency promotes the expression of both isoforms of *Neat1* (Hu et al., 2015). We therefore anticipated that elevating CARM1 levels might have similar consequences for paraspeckles as depleting *Neat1* transcripts. To test this possibility, we overexpressed CARM1 by injecting increasing concentrations of synthetic *Carm1* mRNA into the zygote (Figures 4A and S4A). We found that overexpression of CARM1 indeed reduced the levels of *Neat1* transcript (Figure 4B) and led to disruption of paraspeckles and re-localization of their components, including p54nrb, around the nucleoli in 58%, 61%, and 87% ± 3% (mean ± SD) of analyzed nuclei (depending on the concentration of the synthetic mRNA injected) at the 4-cell stage, just as with *Neat1* depletion (Figures 4C and 4D; Figures S4B and S4C; Figures 3J and 3K). CARM1 overexpression did not change the expression level of p54nrb, as judged by its average staining intensity, but only its localization (Figures 4C and 4E; Figures S4C and S4D). This observation was confirmed when CARM1 was overexpressed in half of the embryo (Figures S4E and S4F).

Because CARM1 has also been shown to methylate p54nrb to regulate its ability to bind certain classes of RNA (Hu et al., 2015), we asked whether loss of this enzymatic activity might affect paraspeckle behavior. We found that when we treated embryos with a CARM1 inhibitor, this also disrupted p54nrb paraspeckles and led p54nrb to form larger clusters or localize around the nucleolus (Figure 4F) with only a 1.2-fold increase of p54nrb expression level, as judged by its average intensity (Figure 4G). Thus, the levels and activity of CARM1 appear to be critical for paraspeckle organization; either overexpression of CARM1 or loss of its activity affects the physical distribution of p54nrb. This suggests that CARM1 contributes to feedback mechanisms that dynamically regulate the formation and nuclear compartmentalization of paraspeckles.

### Depletion of Single Paraspeckle Components Causes Early Developmental Arrest

**Figure 5:**
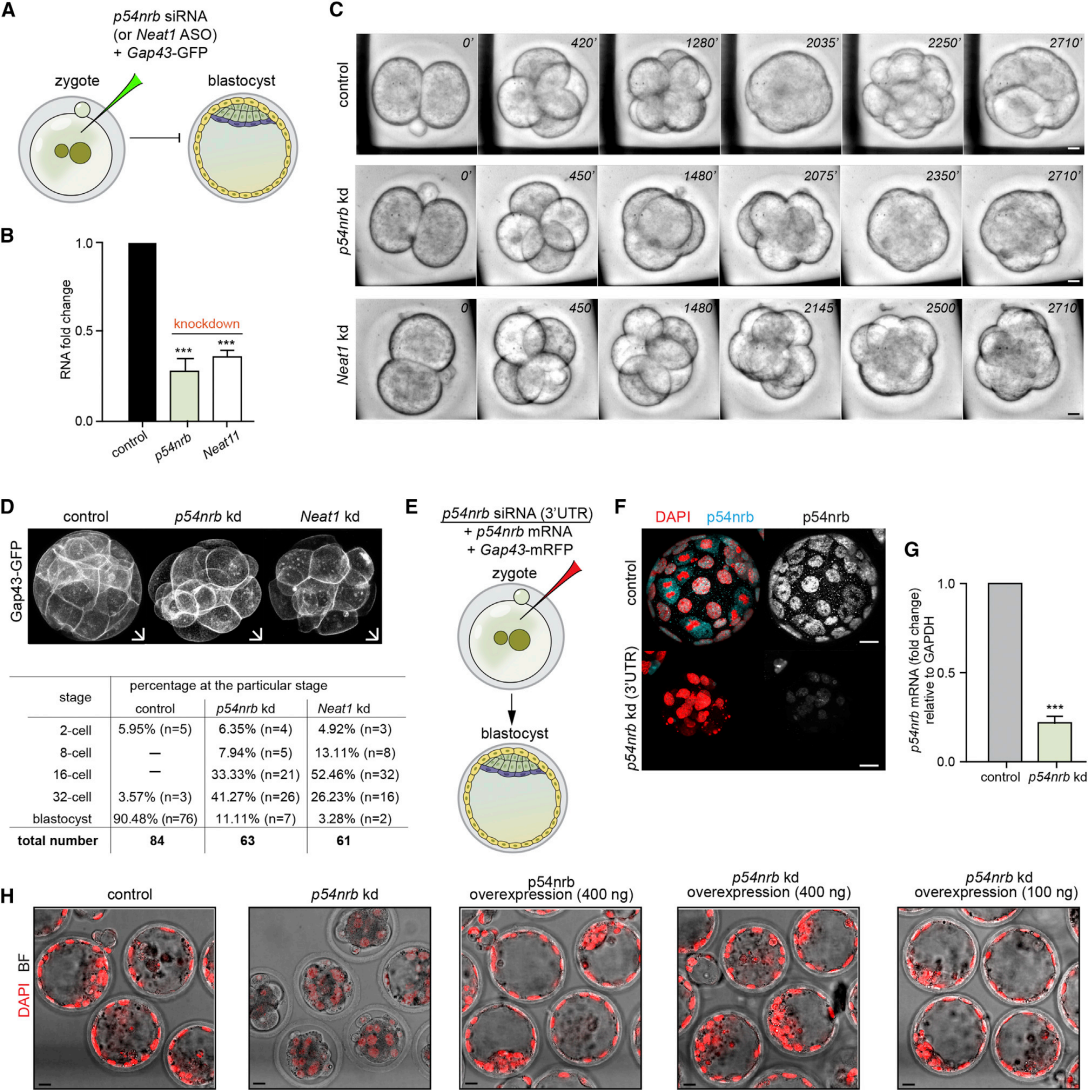
Depletion of Paraspeckle Components Leads to Developmental Arrest at the 16- to 32-Cell Stage (A) Scheme for *p54nrb* siRNA and *Neat1* ASO depletion. Zygotes were injected with either *p54nrb* siRNA (or *Neat1* ASOs) or control siRNA (or control ASOs) and *Gap43*-GFP mRNA. Embryos were either subjected to RNA isolation and qRT-PCR at the 2-cell stage or left to develop in culture. (B) qRT-PCR of 2-cell stage embryos injected with either control siRNA (n = 60, 3 biological replicates), *p54nrb* siRNA (n = 86, 3 biological replicates), or *Neat1* ASO (n = 54, 3 biological replicates), validating *p54nrb* and *Neat1* knockdown efficiency (Student’s t test, p < 0.001). (C) Differential interference contrast (DIC) time-lapse images from the 2-cell stage to 32-cell stage of control (n = 10), *p54nrb*-depleted (n = 12), and *Neat1*-depleted (n = 8) embryos. Scale bars, 10 μm. (D) 3D representations of the control, *p54nrb*-, and *Neat1*-depleted embryos (top). Also shown is a table analyzing the extent of development of embryos in *in vitro* culture (bottom). Scale bars, 10 μm. (E) Scheme for *p54nrb* siRNA and of the rescue experiment. Zygotes were injected with *p54nrb* siRNA directed against its 3′ UTR (n = 26), *p54nrb* synthetic mRNA (n = 31), or co-injected with *p54nrb* siRNA directed against its 3′ UTR along with *p54nrb* synthetic mRNA (n = 54). (F) Confocal images of control and *p54nrb* siRNA-injected embryos (RNAi directed against the 3′ UTR). White, p54nrb; red, DNA (DAPI). Scale bars, 10 μm. (G) qRT-PCR of 2-cell stage embryos injected with either control or *p54nrb* siRNA directed against its 3′ UTR (n = 54, 3 biological replicates), validating *p54nrb* knockdown efficiency (Student’s t test, p < 0.001). (H) Confocal images of embryos injected with control siRNA (n = 20), *p54nrb* siRNA (directed against the 3′ UTR; n = 26), or *p54nrb* mRNA (n = 31) and co-injected with *p54nrb* siRNA along with 100 ng (n = 23) or 400 ng *p54nrb* mRNA (n = 31). Scale bars, 10 μm. Error bars represent SEM.

**Figure S5:**
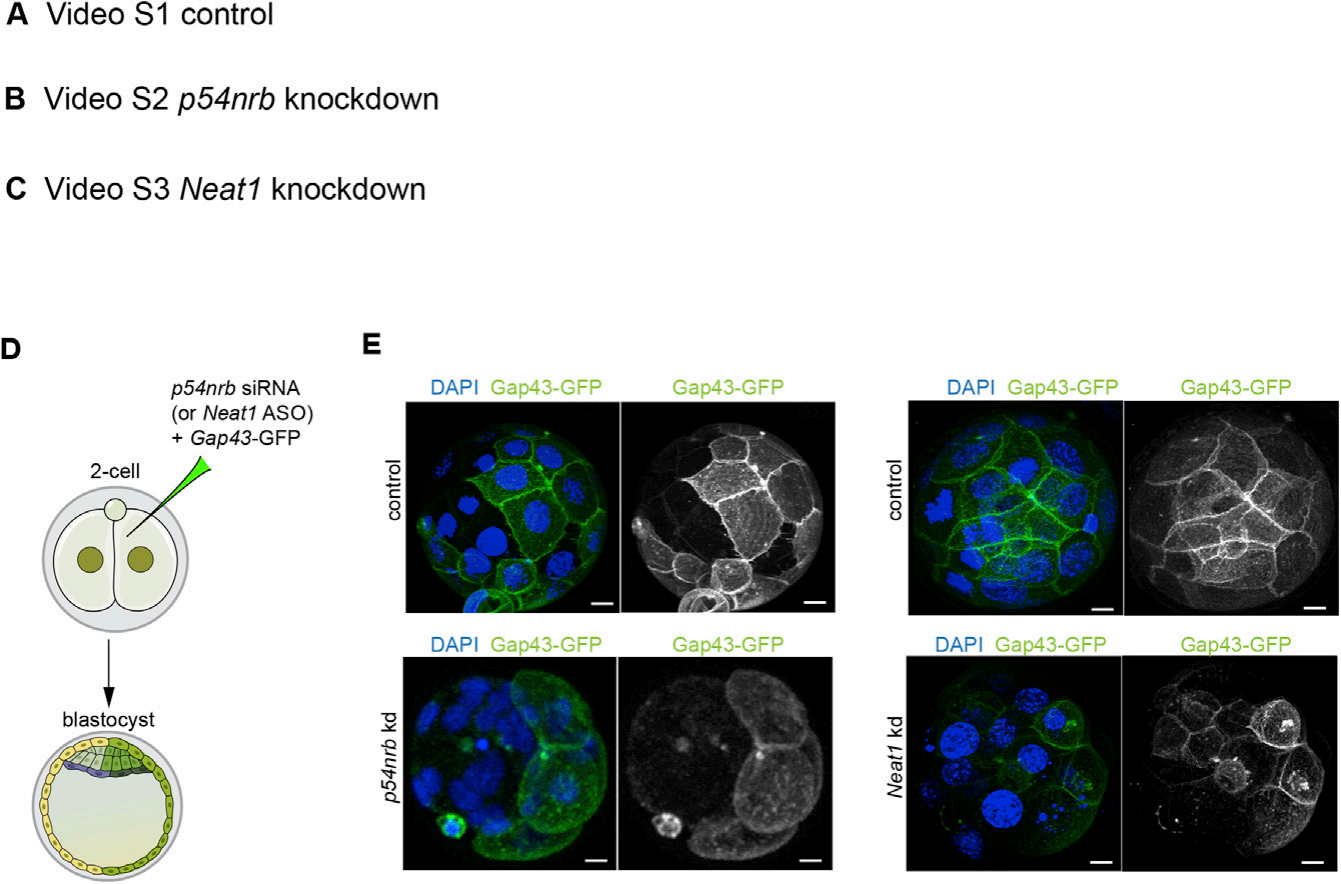
Depletion of *p54nrb* or *Neat1* Leads to Pre-implantation Embryo Arrest, Related to Figure 5 (A,B,C) Time lapse imaging experiment. Following injection with either control (n = 10 embryos) or *p54nrb* siRNAs (n = 12 embryos) or *Neat1* ASOs (n = 8 embryos), embryos were observed on a spinning disc confocal microscope from the 2-cell stage onward. (D) Scheme of *p54nrb* and *Neat1* knockdowns experiment. One blastomere of 2-cell stage embryo was injected with control and *p54nrb* siRNAs or control and *Neat1* ASOs together with *Gap43*-GFP mRNA (injection control). Embryos were cultured until the blastocyst stage and subjected to immunostaining. (E) Confocal images showing development of the injected blastomere; DNA (DAPI, blue) and membrane (Gap43-GFP; green) in control (n = 14), *p54nrb* (n = 15) and *Neat1*-depleted embryos (n = 10) in a representative nucleus from a 4-cell stage embryo. Scale bar 10 μm.

To address the role of paraspeckle components in early development, we used RNAi as an approach to simultaneously downregulate both maternal and zygotic transcripts of *p54nrb* and ASOs to deplete the long form of *Neat1* (Figure 5A). We confirmed with RT-PCR that we could deplete p54nrb and Neat1 transcripts by the 2-cell stage (Figure 5B) and scored the effects of depletion of these individual paraspeckle components on the developmental progression of the embryos. We found that depletion of *p54nrb* and *Neat1* resulted in developmental arrest by the 16-cell (33.33% of embryos, *p54nrb* siRNA; 52.46%, *Neat1* ASOs) and 32-cell stages (41.27% of embryos, *p54nrb* siRNA; 26.23%, *Neat1* ASOs) (Figures 5C and 5D; Videos S1, S2, and S3). To verify this phenotype, we injected a single blastomere of 2-cell stage embryos with *p54nrb* siRNA, *Neat1* ASOs, or control siRNA together with *Gap43*-GFP mRNA (Figure S5D). Depletion of either p54nrb or *Neat1* in one blastomere resulted in developmental arrest of its progeny cells after 4–5 cell division cycles. The control uninjected blastomere developed to the blastocyst stage (Figure S5E). We confirmed that we could also recapitulate this developmental arrest phenotype by injecting siRNA targeting the 3′ UTR of *p54nrb* (Figures 5E and 5F). Successful knockdown by the siRNA was confirmed by RT-PCR (Figure 5G). To address whether we could rescue the effects of the *p54nrb* knockdown, we co-injected RNAi and resistant synthetic mRNA for *p54nrb* into the zygote. This allowed the embryo to develop until the blastocyst stage, indicating that p54nrb overexpression is able to rescue the effects of its depletion and overcome the embryo arrest (Figures 5E and 5H). Together, these experiments suggest that paraspeckle components are required for correct development to the blastocyst stage.

Video S1. Time-Lapse Imaging of Mouse Embryos from the 2-Cell Stage to the 32-Cell Stage of Control Embryos, Related to Figure 5

Video S2. Time-Lapse Imaging of Mouse Embryos from the 2-Cell Stage to the 32-Cell Stage of *p54nrb*-Depleted Embryos, Related to Figure 5

Video S3. Time-Lapse Imaging of Mouse Embryos from the 2-Cell Stage to the 32-Cell Stage of *Neat1*-Depleted Embryos, Related to Figure 5

### CARM1 Is Required for a Subset of Paraspeckle Functions

**Figure 6:**
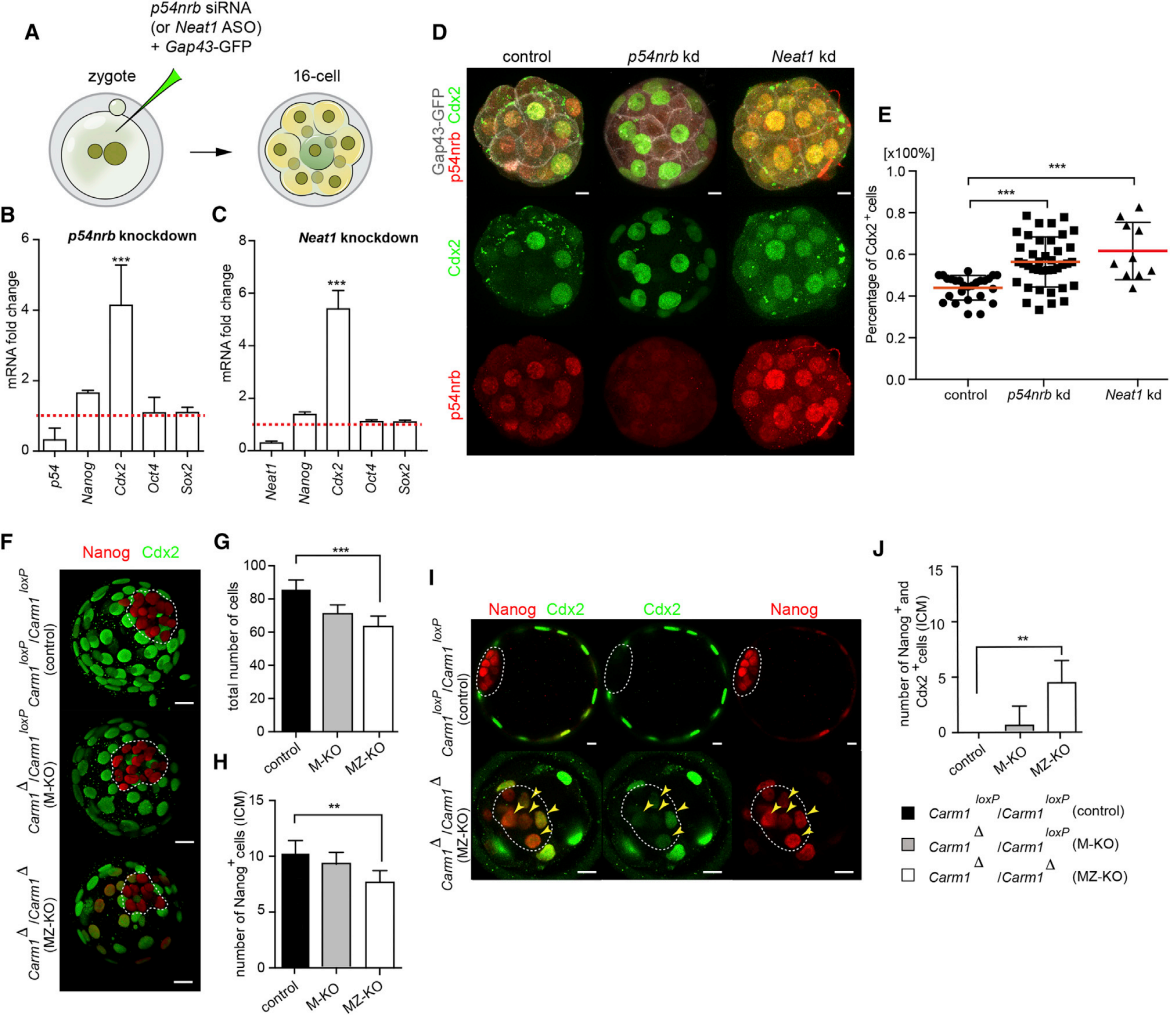
Effects of Depletion of Paraspeckle Components and Loss of CARM1 upon Cdx2 and Nanog Expression (A) Scheme of *p54nrb* siRNA (and *Neat1* ASO) knockdown. Zygotes were injected with *p54nrb* siRNA or control siRNA and *Gap43*-GFP mRNA or with *Neat1* ASO and control ASO and *Gap43*-GFP mRNA. Embryos were cultured until the 16-cell stage and either subjected to RNA isolation and qRT-PCR or immunostaining. (B) qRT-PCR of 16-cell stage embryos injected with either control (n = 54, 3 biological replicates) or *p54nrb* siRNA (n = 90, 3 biological replicates). (C) qRT-PCR of 16-cell stage embryos injected with either control (n = 63, 3 biological replicates) or *Neat1* ASO (n = 75, 3 biological replicates). (D) Confocal images of Cdx2 and p54nrb expression in embryos injected with control, *p54nrb* siRNA, or *Neat1* ASOs. Gap43-GFP (white) was used as a marker of injection and labels the membrane. Scale bars, 10 μm. (E) Quantification of the number of Cdx2^+^ cells in control (n = 32), *p54nrb*-depleted (n = 42), and *Neat1*-depleted embryos (n = 10) at the 16- 32-cell stage (ANOVA test, p < 0.0001). (F) 3D reconstruction of confocal images of Cdx2 and Nanog expression in *Carm1*^loxP^/*Carm1*^loxP^ (control), *Carm1*Δ/*Carm1*^loxP^ (M-KO), and *Carm1*Δ/*Carm1*Δ (MZ-KO). Dashed lines mark the ICM. Scale bars, 10 μm. (G) Quantification of the total number of cells in *Carm1*^loxP^/*Carm1*^loxP^ (control, n = 10), *Carm1*Δ/*Carm1*^loxP^ (M-KO, n = 8), and *Carm1*Δ/*Carm1*Δ (MZ-KO, n = 10) embryos (ANOVA test, p < 0.0001). (H) Quantification of Nanog^+^ cells in the ICM of *Carm1*^loxP^/*Carm1*^loxP^ (control), *Carm1*Δ/*Carm1*^loxP^ (M-KO), and *Carm1*Δ/*Carm1*Δ (MZ-KO) embryos (ANOVA test, p < 0.001). (I) Confocal images showing Cdx2- and Nanog-positive cells in control (n = 10) and *Carm1*Δ/*Carm1*Δ (MZ-KO, n = 10). Dashed lines mark the ICM. Scale bars, 10 μm. (J) Quantification of Nanog^+^ and Cdx2^+^ double-positive cells in the ICM of *Carm1*^loxP^/*Carm1*^loxP^ (control, n = 10), *Carm1*Δ/*Carm1*^loxP^ (M-KO, n = 8), and *Carm1*Δ/*Carm1*Δ (MZ-KO, n = 10) embryos (ANOVA test, p < 0.001). Error bars represent SEM.

The developmental phenotypes resulting from disruption of paraspeckles led us to determine the effects of depleting p54nrb upon expression of genes regulating early development, including lineage markers of TE (*Cdx2*) and ICM (*Sox2, Nanog*, and *Oct4*). We found that when we depleted either p54nrb or *Neat1*, the levels of *Sox2*, *Oct4*, or *Nanog* were not significantly affected at the 16-cell stage of development. However, *Cdx2* mRNA levels increased by 4.0- to 5.0-fold (Figures 6A–6C). Associated with this, we found that depletion of p54nrb or *Neat1* led to a 2-fold increase in the number of cells expressing Cdx2 protein (Figures 6D and 6E).

These results would accord with the role of paraspeckles to facilitate the activity of CARM1, which has been shown to stimulate expression of *Sox2* and *Nanog* and drive cells toward a pluripotent fate (Torres-Padilla et al., 2007, White et al., 2016). It further suggests that, in the absence of paraspeckle function, the sequence of events that establish the formation of the pluripotent lineage is not correctly executed. As a consequence, development arrests around the 16- 32-cell stage.

**Figure S6:**
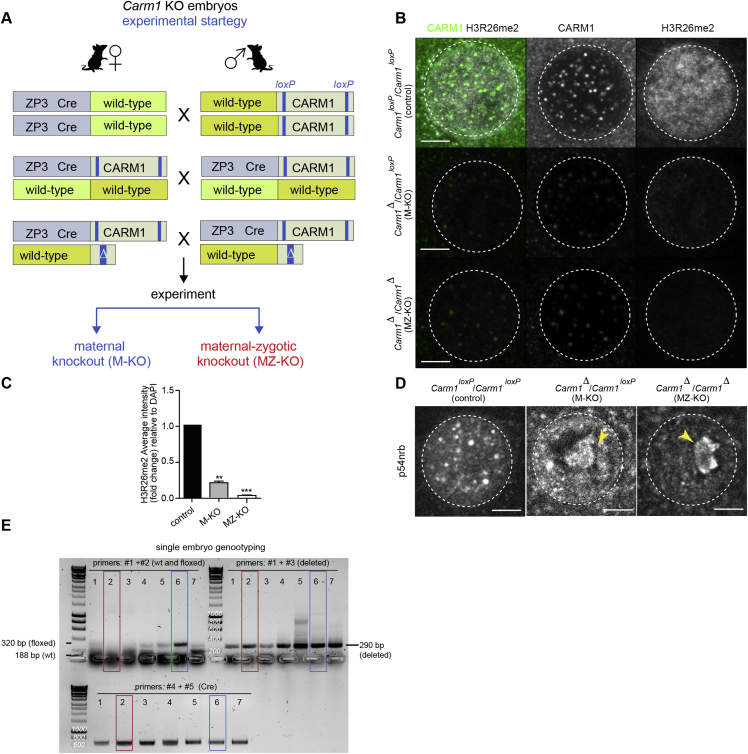
Generation of Maternal and Maternal-Zygotic *Carm1* Knockouts, Related to Figure 6 (A) Schematic representation of the experimental strategy to generate maternal (M-KO) and maternal-zygotic (MZ-KO) knockouts to remove the active pool of CARM1. (B) Confocal images of CARM1 (green) and H3R26me2 (gray) expression in *Carm1*^loxP^/*Carm1*^loxP^ (control), *Carm1*Δ/*Carm1*^loxP^ (M-KO) and *Carm1*Δ/*Carm1*Δ (MZ-KO showing representative nuclei from 4-cell stage embryos. Scale bar 5 μm. (C) Quantification of relative fluorescence intensity of H3R26me2 in *Carm1*^loxP^/*Carm1*^loxP^ (control; n = 12), *Carm1*Δ/*Carm1*^loxP^ (M-KO; n = 8) and *Carm1*Δ/*Carm1*Δ (MZ-KO;n = 12) (Mann-Whitney test, ^∗∗^p = 0.0022 and ^∗∗∗^p = 0.0007). (D) Confocal images of p54nrb expression in *Carm1*^loxP^/*Carm1*^loxP^ (control), *Carm1*Δ/*Carm1*^loxP^ (M-KO) and *Carm1*Δ/*Carm1*Δ (MZ-KO) showing representative nuclei from 4-cell stage embryos. Yellow arrowheads indicate p54nrb clusters. Scale bar 5 μm. (E) DNA electrophoresis of *Carm1*^loxP^/*Carm1*^loxP^ (control), *Carm1*Δ/*Carm1*^loxP^ (M-KO) and *Carm1*Δ/*Carm1*Δ (MZ-KO) embryos. Red brackets indicate MZ-KO and blue brackets correspond to M-KO *Carm1* embryos. Error bars represent s.e.m.

Knowing that reduction of p54nrb levels both in embryos and ESCs also affects CARM1 expression and histone H3R26 methylation, it might be anticipated that knockout embryos, deficient in both maternal and zygotic CARM1 protein, might also arrest at the 16- 32-cell stage of development. This would be at an earlier stage of development than reported following inhibition of CARM1 from the 2-cell stage by inhibitor or its depletion by RNAi (Panamarova et al., 2016, Goolam et al., 2016, White et al., 2016). We therefore used a genetic approach to deplete maternal and zygotic pools of CARM1 from the oocyte stage onward (Figure S6A). The knockout embryos showed a dramatic reduction in the numbers and intensity of CARM1 speckles and of the R26-methylated form of histone H3, which was almost completely eliminated in embryos missing both maternal and zygotic expression of CARM1 (Figures S6B and S6C). As we had found following *Neat1* depletion or treatment with the CARM1 inhibitor, we observed aggregation of p54nrb around the nucleolus in maternal and zygotic *Carm1* knockout embryos (*Carm1* MZ-KO) at the 4-cell stage (Figure S6D). Although they did not arrest in development as early as *Neat1*- or *p54nrb*-depleted embryos, the *Carm1* MZ-KO embryos were delayed in their development and formed blastocysts with reduced numbers of cells in which lineage specification had not occurred correctly (Figures 6F–6I). We analyzed development of 19 *Carm1* MZ-KO embryos and 15 of their heterozygous *Carm1* maternal-only knockout (M-KO) siblings (Figure S6E). This revealed that the absence of both maternal and zygotic *Carm1* led to a decreased total number of cells compared with embryos with the maternal depletion alone or wild-type embryos (Figure 6H). Moreover, the number of ICM cells expressing Nanog was significantly lower in *Carm1* MZ-KO embryos compared with control wild-type embryos analyzed at the same developmental stage. The *Carm1* MZ-KO embryos also exhibited cases in which there was both ectopic expression of Nanog in outside cells (Figures 6F and 6G) and co-expression of Cdx2 and Nanog in the ICM (Figures 6I and 6J). These results accord with a role of CARM1 in assigning the fate of the pre-implantation lineages. Together, the results we present here show that, although loss of *Neat1* or *Carm1* each results in re-localization of p54nrb from paraspeckles to a peri-nucleolar position, the developmental phenotype of *Carm1* MZ-KO embryos is less severe than in embryos following knockdown of *Neat1* and *p54nrb*, suggesting that p54nrb and *Neat1* have additional roles earlier in embryo development beyond the functions of CARM1.

## Discussion

In this study, we demonstrated that CARM1 accumulates in nuclear speckles by the 4-cell stage of mouse embryo development and that the number of speckles varies between the four cells in accord with the level of methylation of the CARM1 substrate, histone H3R26, shown previously to correlate with a bias toward the specification of a pluripotent cell fate (Torres-Padilla et al., 2007; reviewed in Chen et al., 2018).

**Figure 7:**
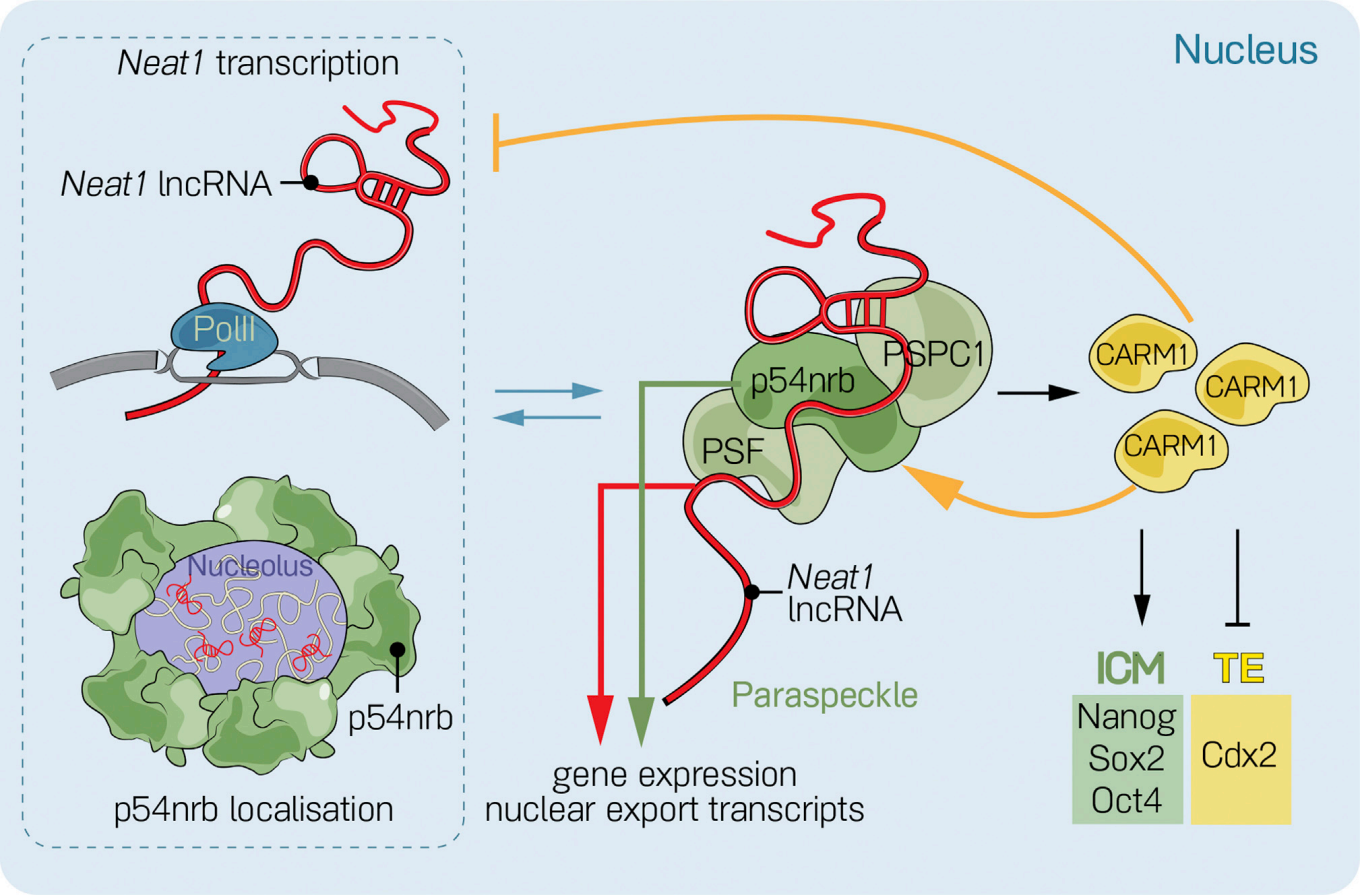
Model of the Dependency of Paraspeckle Assembly and Function on CARM1 Levels The assembly and structure of paraspeckles rely on constitutive transcription of its seeding lncRNA, *Neat1*. In the 2- and 4-cell stage embryo, *Neat1* recruits paraspeckle proteins, including CARM1, to the paraspeckle structure. This sets up a negative feedback loop because of the inhibitory effect of CARM1 on *Neat1* transcription. As a consequence, *Neat1*’s partner, p54nrb, localizes around the periphery of the nucleolus either when *Neat1* is depleted or when CARM1 is overexpressed. Optimal levels of the active form of CARM1 are critical for the function and localization of p54nrb, a CARM1 substrate. Thus, we hypothesize that inhibition or loss of CARM1 results in aggregation of p54nrb by preventing its correct function as part of a positive feedback loop. Changes in the functional organization of paraspeckles through depletion of either *Neat1* or p54nrb perturb the expression and function of genes involved in establishing the embryonic (ICM) and extra-embryonic (TE) lineages, including CARM1. Loss of CARM1 downstream of *Neat1* and p54nrb affects the expression of pluripotency genes, as shown previously.

About two-thirds of the CARM1 speckles correspond to paraspeckles, as judged by the presence of the *bona fide* paraspeckle components p54nrb and PSPC1 proteins and *Neat1* lncRNA. *Neat1* RNA seeds paraspeckle formation by sequestering dimers of p54nrb and SFPQ (splicing factor, proline- and glutamine-rich) (Fox et al., 2002, Fox et al., 2005). Thus, our finding that depletion of *Neat1* RNA results in perturbation of paraspeckles, changed nuclear distribution of p54nrb, and failure to recruit CARM1 reflects the key role of *Neat1* in establishing paraspeckles. Similarly, depletion of p54nrb leads to a similar reduction of CARM1 speckles and reduced methylation of histone H3R26. Together, this accords with a requirement of the assembled paraspeckle as a body able to recruit CARM1 and promote its function. Our results also suggest that CARM1 might feed back and modulate paraspeckle formation in at least two ways. First, we find that elevating CARM1 levels has a similar consequence as depletion of *Neat1,* a finding that accords with the ability of CARM1 to interact with the *Neat1* promoter and repress *Neat1* expression (Hu et al., 2015). This suggests a model whereby *Neat1* promotes the assembly of paraspeckles that recruit CARM1, which can then feed back and inhibit transcription of *Neat1* and so regulate paraspeckle number (Figure 7). Second, we find that inhibiting CARM1 also leads to redistribution of p54nrb. This suggests that CARM1 activity is required for the organization of paraspeckles and accords with its ability to methylate and modulate the function of p54nrb (Hu et al., 2015). This potential ability of CARM1 to provide negative and positive feedback suggests that precise CARM1 levels might be critical for optimum assembly of paraspeckles (Figure 7).

However, our results indicate that an inability of paraspeckles to form in the absence of CARM1 would not completely eliminate their function because the phenotype resulting from CARM1 depletion or inhibition is less severe than that seen following elimination of either *Neat1* or p54nrb. This suggests that the aggregate of p54nrb seen around the nucleolus, when CARM1 levels are perturbed, might retain some functionality. Paraspeckles are increasingly viewed as liquid droplets within the nucleus that permit sequestering of RNAs and proteins through phase separation (Yamazaki et al., 2018, Fox et al., 2018). In this context, it is possible that the peri-nucleolar aggregate of p54nrb and the paraspeckle are two manifestations of similar membrane-less organelles whose structure reflects their precise components. If this is the case, then CARM1 might be one client protein of paraspeckles that, when incorporated, can also influence paraspeckle organization. Our findings suggest that the CARM1 concentration influences the equilibrium of the assembly and disassembly of paraspeckles. It is possible to speculate that the concentration of CARM1 within the phase-separated nuclear granules could enhance and/or coordinate biological reactions, such as histone H3R26 methylation, and so contribute to the differential properties of 4-cell blastomeres observed previously (Torres-Padilla et al., 2007, Goolam et al., 2016, White et al., 2016). Changes in CARM1 concentration might also regulate the physical properties of nucleoplasm by tuning the phase separation of paraspeckles and molecules associated with them. In this way, CARM1 might enhance molecular crowding and provide a driving force to shape nuclear compartmentalization (Rea et al., 2000). Alternatively, the molecular condensation and formation of paraspeckles may function to sequester factors not required at the particular developmental stage and prevent their inappropriate action.

Disruption of CARM1’s nuclear distribution by depleting *Neat1* or p54nrb led to its reduced ability to methylate histone H3R26, disproportionate expression of the TE transcription factor Cdx2, and developmental arrest at the 16- to 32-cell stage without correctly undertaking the first cell fate decision. Although we focused here on the effects of CARM1 disruption on Cdx2, because of its role as master regulator of differentiation into the first extra-embryonic lineage, we cannot exclude the possibility that CARM1 has other roles. We observed that *Carm1* knockout embryos, deficient for both maternal and zygotic protein, also showed delayed development and formed small blastocysts with fewer Nanog expressing inside cells. These embryos also had cells in which fate decisions had not occurred correctly: outside cells expressing Nanog and inside cells co-expressing Nanog and Cdx2. Thus, the development of genetic knockout embryos is delayed compared with embryos in which CARM1 was downregulated by RNAi, which also resulted in blastocysts with decreased numbers of Nanog-expressing inside cells (White et al., 2016, Goolam et al., 2016), possibly reflecting less effective CARM1 depletion by RNAi. These phenotypes can be accounted for by reduced H3R26me2 (Torres-Padilla et al., 2007, Goolam et al., 2016, White et al., 2016; this study), which regulates the accessibility of chromatin to stimulate development of pluripotent tissue (White et al., 2016). The failure of pluripotent tissue to develop following disruption of paraspeckles by *Neat1* or *p54nrb* depletion would accord with a requirement for CARM1 for this developmental process. *Neat1* has been shown to associate with hundreds of active chromatin sites in cultured human cells (West et al., 2014), and its localization on active chromatin sites responds to changes in transcription and interactions with proteins resident in nuclear bodies. Thus, *Neat1* could link CARM1 to nuclear subdomains and active chromatin sites. CARM1 can act as a co-suppressor (Xu et al., 2001) and a co-activator (Chen et al., 1999, Bedford and Clarke, 2009) for a number of transcription factors. This is pertinent to the pre-implantation mouse embryo, where CARM1-mediated H3R26me2 has been shown to correlate with cell fate choice (Torres-Padilla et al., 2007). Moreover, it has been shown recently that Sox21 expression and, consequently, regulation of Cdx2 transcription significantly depend on CARM1 expression and H3R26 methylation (Goolam et al., 2016). The more severe phenotype resulting from *Neat1* or p54nrb depletion compared with CARM1 depletion suggests a requirement either for paraspeckles per se and/or their individual components in regulating a more extensive set of early developmental processes than mediated by CARM1 alone. This outcome may also be accentuated by the known redundancy of CARM1, whose function can be substituted by Prdm14, a chromatin modifier that, when overexpressed, can also increase the level of H3R26me2 and predispose their progeny to the pluripotent lineage (Burton et al., 2013).

Importantly, *Neat1* is one of the few lncRNAs having a robust phenotype when knocked out in mice, compromising the secretory function and the development of critical tissues relating to female reproduction (Nakagawa et al., 2014, Standaert et al., 2014). It is likely that, at later developmental stages, *Neat1* functions could be carried out by a compensatory gene in several tissues (Rossi et al., 2015). The best current candidate for such a gene is *Malat1*, which encodes a scaffold lncRNA for nuclear speckles. Indeed, it has been demonstrated that *Malat1* knockout mice show elevated *Neat1* expression, suggesting some redundancy of function (Nakagawa et al., 2012, Zhang et al., 2012, West et al., 2014). The maternal effect in *Neat1* knockout mice was proposed to be due to altered *corpus luteum* function, but notably, embryo development was not examined in these studies (Nakagawa et al., 2011). Our findings indicate that depletion of maternal and zygotic *Neat1* RNA from the zygote leads to developmental arrest at the 16- to 32-cell stage. This requirement for *Neat1* in early development receives support from our finding that depletion of its paraspeckle partner, p54nrb, also results in arrest at the 16- to 32-cell stage, in agreement with embryonic lethality of the *p54nrb* knockout (Cox et al., 2010). The ability to rescue embryogenesis blocked as a result of RNAi directed against the UTR of *p54nrb* mRNA by injecting synthetic mRNA for *p54nrb* that had an alternative UTR validates the specificity of the knockdown. Together, our results suggest that the *Neat1*-p54nrb complex plays roles at this stage of pre-implantation development. Other pointers to the function of p54nrb in embryogenesis have come from findings with embryoid bodies generated from the *p54nrb* knockout ESC cells, which were much smaller and displayed disorganized structures compared with wild-type controls (Ma et al., 2016). In addition to the compromised activation of some developmental genes, such as *Cdx2* and *Sox17*, *p54nrb* knockout cells also showed compromised repression of the pluripotent genes, including *Nanog*, *Sox2*, and *Oct4* (Ma et al., 2016). Together, these results point to a potential function of paraspeckle components as regulatory hubs of gene expression in the early embryo associated with accurate lineage allocation.

It has been proposed that differences between cells in the mouse embryo do not arise until the 16-cell stage, when cells acquire inside or outside positions (Hiiragi and Solter, 2004, Alarcón and Marikawa, 2005, Motosugi et al., 2005). Although the ability to differentiate into either ICM or TE can be retained until the 16-cell stage (Tarkowski and Wróblewska, 1967, Suwińska et al., 2008), growing evidence suggests that heterogeneity that biases cell fate is already established by the 4-cell stage (Piotrowska et al., 2001, Gardner, 2001, Piotrowska-Nitsche and Zernicka-Goetz, 2005, Fujimori et al., 2003, Plachta et al., 2011, Tabansky et al., 2013). This early heterogeneity in cell fate has been tied to variation in the levels of histone H3R26 methylation by CARM1 (Torres-Padilla et al., 2007) and Prdm14 (Burton et al., 2013) leading to increased Sox2-DNA binding (White et al., 2016) and resulting in upregulation of Sox2-dependent genes linked to pluripotency (Goolam et al., 2016). The association of CARM1 with paraspeckles that number also varies at the 4-cell stage, offers one possible way by which cells can assert differential developmental properties.

The roles of paraspeckles in mouse embryo development might reflect the variety of suggested mechanisms through which they might regulate gene expression. The direct interactions of paraspeckle components with chromatin and enrichment of p54nrb-bound genes in the category of transcription regulation and developmental genes, including *Cdx2* (Bernstein et al., 2006, Vastenhouw and Schier, 2012) supports a role for paraspeckle components at the transcriptional level. Paraspeckles have also been proposed to titrate specific mRNAs by sequestering them within the nucleus, from where they are not released for translation until *Neat1* is degraded or downregulated (Prasanth and Spector, 2007, Ip and Nakagawa, 2012, Chen and Carmichael, 2009), a regulatory process in which CARM1 has been proposed to participate (Hu et al., 2015). It seems likely that a combination of mechanisms allows paraspeckles to regulate the expression and function of developmentally important transcripts, including Cdx2. Our findings allow us to conclude that *Neat1* and p54nrb provide a scaffolding function in the nucleus and that they contribute to the regulation of embryo development by modulating the expression of lineage-specifying genes either directly, by regulating their transcription or by binding to their products, or indirectly, by acting through CARM1. Together, our results bring a novel perspective of the nuclear architecture of individual cells in the embryo during development and lineage allocation in which CARM1 is linked to the paraspeckles. They also pave the way toward understanding how the spatial and nuclear organization can modulate gene expression and, subsequently, affect developmental processes.

## STAR★Methods

### Key Resources Table

**Table:** 

REAGENT or RESOURCE	SOURCE	IDENTIFIER
**Antibodies**		

mouse monoclonal anti-CARM1 (3H2)	Cell Signaling	Cat# 12495
rabbit polyclonal anti-CARM1	Cell Signaling	Cat# 4438; RRID:AB_2068436
rabbit polyclonal anti-CARM1	Bethyl Laboratories	Cat# A300-421A; RRID:AB_2068452
rabbit polyclonal anti-CARM1	Active Motif	Cat# 39251
mouse monoclonal anti-Cdx2	Biogenex	Cat# MU392-UC; RRID:AB_2335627
mouse monoclonal anti-GAPDH	Santa Cruz Biotechnology	Cat# sc-47724; RRID:AB_627678
rat monoclonal anti-GFP	Nacalai Tesque	Cat# 04404-84; RRID:AB_10013361
rabbit polyclonal anti-H3	Abcam	Cat# ab18521; RRID:AB_732917
rabbit polyclonal anti-H3R26me2	Abcam	Cat# ab127095; RRID:AB_2732841
rabbit polyclonal anti-Nanog	Abcam	Cat# 80-892; RRID:AB_2150114
mouse monoclonal anti-Oct3/4	Santa Cruz Biotechnology	Cat# sc-5279; RRID:AB_628051
rabbit polyclonal anti-p54nrb [EPR5270]	Abcam	Cat# ab133574
goat polyclonal anti-p54nrb	Santa Cruz Biotechnology	Cat# sc-23249; RRID:AB_653376
rabbit polyclonal anti-PSF	Santa Cruz Biotechnology	Cat# sc-28730; RRID:AB_2186937
rabbit polyclonal anti-PSPC1	Santa Cruz Biotechnology	Cat# sc-84577; RRID:AB_2171459
goat polyclonal anti-Sox17	R&D Systems	Cat# AF1924; RRID:AB_355060
mouse monoclonal anti-tubulin	Sigma	Cat# T5168; RRID:AB_477579
secondary horseradish peroxidase-conjugated goat anti-mouse IgG (H+L)	Jackson ImmunoResearch	Cat# 115-035-003; RRID:AB_10015289
secondary horseradish peroxidase-conjugated goat anti-rabbit IgG (H+L)	Jackson ImmunoResearch	Cat# 111-035-144; RRID:AB_2307391
Alexa Fluor 488 donkey anti-mouse IgG (H+L)	Thermo Fisher Scientific	Cat# R37114; RRID:AB_2556542
Alexa Fluor 568 donkey anti-goat IgG (H+L)	Thermo Fisher Scientific	Cat# A-11057; RRID:AB_2534104
Alexa Fluor 647 donkey anti-rabbit IgG (H+L)	Thermo Fisher Scientific	Cat# A-31573; RRID:AB_2536183
Alexa Fluor 488 donkey anti-rabbit IgG (H+L)	Thermo Fisher Scientific	Cat# A-21207; RRID:AB_141637
Alexa Fluor 568 donkey anti-mouse IgG (H+L)	Thermo Fisher Scientific	Cat# A-10037; RRID:AB_2534013
Alexa Fluor 647 donkey anti-mouse IgG (H+L)	Thermo Fisher Scientific	Cat# A-31571; RRID:AB_162542

**Bacterial and Virus Strains**		

DH5α Competent Cells	Invitrogen/Fisher Scientific	LS18265017

**Chemicals, Peptides, and Recombinant Proteins**		

Acrylamide	SevernBiotech Ltd	Cat# 20-2100-10
β-mercaptoethanol	Thermo Fisher Scientific	Cat# 31350-010
BSA	Sigma-Aldrich	Cat# A2153
CARM1 inhibitor	Millipore	Cat# 217531
DAPI	Sigma-Aldrich	Cat# D9542
Denhardt’s solution	Sigma-Aldrich	Cat# 30915
Dextran sulfate	Sigma-Aldrich	Cat# D8906
Dimethyl sulfoxide (DMSO)	Sigma-Aldrich	Cat# D8414
Dulbecco’s modified Eagle’s medium (DMEM)	Thermo Fisher Scientific	Cat# 41966
Fetal Bovine Serum (FCS)	Stem Cell Institute, Cambridge, UK	N/A
Formamide	Sigma-Aldrich	Cat# F9037
GlutaMAX	Thermo Fisher Scientific	Cat# 35050061
HiPerFect	QIAGEN	Cat# 3011704
KSOM	Millipore	Cat# MR-020P-5F
Lipofectamine RNAiMax	Invitrogen	Cat# 13778030
MEM non-essential amino acids	Thermo Fisher Scientific	Cat# 11140035
Mineral oil for *in vitro* embryo culture	Life Global	Cat# CE0086
Mitomycin C	Sigma-Aldrich	Cat# M4287
Paraformaldehyde (PFA)	Fisher Scientific	Cat# AA433689L
Phosphatase Inhibitor Cocktail	Thermo Scientific	Cat# 88667
Protease Inhibitor Cocktail	Roche	Cat# 11836153001
Sodium pyruvate	Thermo Fisher Scientific	Cat# 11360070
20xSSC	Thermo Fisher Scientific	Cat# AM9763
Triton X-100	Sigma-Aldrich	Cat# T8787
Tween	Sigma-Aldrich	Cat# P1379
Tyrode’s solution	Sigma-Aldrich	Cat# T1788
Vanadyl ribonucleoside complex	Sigma-Aldrich	Cat# 94742

**Critical Commercial Assays**		

BCA Protein Assay Kit	Pierce	Cat# 23227
PLA Assay (Duolink)	Sigma-Aldrich	Cat# DUO92102; Cat# DUO92013; Cat# DUO92004; Cat# DUO92002
ECL western blotting substrate	Pierce	Cat# 32109
mMessage mMachine T3 kit	Ambion	Cat# AM1348
PicoPure RNA isolation kit	Thermo Fisher Scientific	Cat# KIT0204
Power SYBR Green RNA-to-CT 1-Step Kit	Thermo Fisher Scientific	Cat# 4389986

**Experimental Models: Cell Lines**		

mESCs (E14)	This paper	N/A
mouse embryonic fibroblasts (MEFs)	Stem Cell Institute, Cambridge, UK	N/A

**Experimental Models: Organisms/Strains**		

Mice C57Bl6/xCBA	Charles River	Strain Code #027
Mice *Carm1 loxP* line	Yadav et al., 2003	https://doi.org/10.1073/pnas.1232272100http://www.pnas.org/content/100/11/6464.long
Mice *Carm1 loxP*/Δ	This paper	N/A
Embryos *Carm1* KO	This paper	N/A

**Oligonucleotides**		

primers for qPCR	Sigma Aldrich	See Table S1
*p54nrb* siRNA	QIAGEN	See Table S1
*Carm1* stealth siRNA	Invitrogen	See Table S1
*Neat1* ASO	Exiqon	See Table S1
Non-specific AllStars Negative Control siRNA	QIAGEN	Cat# SI03650318
Antisense LNA GapmeR negative control	Exiqon	Cat# 1027281

**Recombinant DNA**		

CARM1	Torres-Padilla et al., 2007	https://doi.org/10.1038/nature05458https://idp.nature.com/authorize?response_type=cookie&client_id=grover&redirect_uri=https%3A%2F%2Fwww.nature.com%2Farticles%2Fnature05458
Gap43-RFP	Torres-Padilla et al., 2007	https://doi.org/10.1038/nature05458https://idp.nature.com/authorize?response_type=cookie&client_id=grover&redirect_uri=https%3A%2F%2Fwww.nature.com%2Farticles%2Fnature05458
Gap43-GFP	Torres-Padilla et al., 2007	https://doi.org/10.1038/nature05458https://idp.nature.com/authorize?response_type=cookie&client_id=grover&redirect_uri=https%3A%2F%2Fwww.nature.com%2Farticles%2Fnature05458
p54nrb cDNA	Dharmacon	Cat# MRN1768-202783456

**Software and Algorithms**		

Icy	Open source image analysis software	http://icy.bioimageanalysis.org
ImageJ, Fiji	NIH; Open source image processing software (Schindelin et al., 2012)	https://imagej.nih.gov/ij/index.html
Illustrator CS5	Adobe	N/A
Prism, v7.0a	GraphPad Software Inc	https://www.graphpad.com/scientific-software/prism/

### Contact for Reagent and Resorce Sharing

Further information and requests for resources and reagents should be directed to and will be fulfilled by the Lead Author, Magdalena Zernicka-Goetz (mz205@cam.ac.uk).

### Experimental Model and Subject Details

#### Animals

Animals were maintained in accordance with national and international guidelines. All animal experiments were performed in compliance with Home Office regulations. This research has been regulated under the Animals (Scientific Procedures) Act 1986 Amendment Regulations 2012 following ethical review by the University of Cambridge Animal Welfare and Ethical Review Body (AWERB).

#### Creation of maternal zygotic CARM1 knockout

To obtain oocytes depleted of maternal *Carm1*, females heterozygous for a floxed *Carm1* gene and a *Carm1* deletion (*Carm1Δ*), and carrying a *ZP3-Cre* transgene were used (*Carm1*^*loxP*^*/Carm1Δ*; *ZP3-Cre* females). These females were mated with *Carm1*^*loxP*^*/Carm1Δ* males. The *Carm1*^*loxP*^ line was a kind gift from Mark Bedford (Yadav et al., 2003). Complete cleavage of the *Carm1*^*loxP*^ allele in the female germline by the Cre recombinase results in 100% oocytes carrying the *Carm1Δ*. 50% of the resulting embryos that are *Carm1*Δ*/Carm1***Δ** will be maternal-zygotic (MZ) k-outs and the 50% *Carm1Δ*/*Carm1*^*loxP*^ embryos that have a wild-type (but floxed) paternal *Carm1* allele are heterozygous maternal k-outs. The following set of primers was used to genotype *Carm1* knockout (KO) embryos: #1: 5′AGTTGGTGACCCTTGTGTCC3′; #2: 5′AGCTGCCAGGACCTCTGATA3′; #3: 5′CCTGAGGCAGAAAACAGTATGA3′; #4: 5′GCAGAACCTGAAGATGTTCGC3′; #5: 5′AGGTATCTCTGACCAGAGTCA3′. The combination of #1 and #2 primers detects two bands of 188 bp and 322 bp sizes corresponding to wild-type and floxed allel respectively. Combination of #1 and #3 primers was used to detect a deletion in *Carm1* floxed allele (the expected size of band after deletion 280 bp). Primers #4 and #5 were used in a combination to detect Cre recombinase.

#### Embryo collection

Embryos were collected from 4-6-week-old F1 (C57Bl6xCBA) superovulated female mice with 7.5 IU of pregnant mares’ serum gonadotropin (PMSG; Intervet) and 7.5 IU human chorionic gonadotropin (hCG; Intervet) 48 hours later, and crossed with F1 males. Embryos were isolated in M2 medium supplemented with 4% BSA and cultured in KSOM medium and fixed at the following times post hCG injection: early one-cell stage (19 h post hCG), late one-cell stage (30 h post hCG), early two-cell stage (39 h post hCG), late two-cell stage (48 h post hCG), early four-cell stage (54 h post hCG), late four-cell stage (62 h post hCG), early eight-cell stage (68 h post hCG), late eight-cell stage (74 h post hCG), and early blastocyst stage (98 h post hCG). The zona pellucida was removed using Tyrode’s solution. The following CARM1 inhibitor was used: 1-bezyl-3,5-bis(3-bromo-4-hydroxybenzylidene) piperidin-4-one (7g) (Cheng et al., 2011). Inhibitor was added to 2-cell stage embryos for 10 h at a concentration of 7 μM in KSOM. The inhibitor was dissolved in DMSO. DMSO diluted in KSOM to an equivalent volume to the highest concentration of inhibitor was used for controls.

#### Cell culture

Mouse embryonic stem cells (mESC) E14 were cultured on mitomycin C-treated mouse embryonic fibroblasts (MEFs) in Fc medium comprising DMEM (Dulbecco’s modified Eagle’s medium) supplemented with 15% fetal bovine serum, 2mM GlutaMAX, MEM non-essential amino acids, sodium pyruvate and 100 μM β-mercaptoethanol.

### Method Details

#### Microinjection of pre-implantation embryos

Zygotes from superovulated and mated F1 females were isolated 20 h post-hCG injection. To overexpress p54nrb or CARM1-WT embryos were microinjected with a synthetic mRNA (50 ng μl^−1^, 100 ng μl^−1^ or 400 ng μl^−1^) into the cytoplasm between 24 and 27 h after hCG injection, using an Eppendorf micromanipulator on a Leica inverted microscope. As marker of injection either *Gap43-*GFP or *Gap43-RFP* mRNA (200 ng μl^−1^) were used. Embryos were fixed at indicated times and assessed by immunofluorescence. To deplete p54nrb, CARM1 or *Neat1*, embryos were injected at the zygote stage with a combination of three siRNAs at a total concentration of 12 μM (for p54nrb depletion) 200nM stealth siRNA (for CARM1 depletion) or 200 nM antisense oligonucleotides ASO (for *Neat1* depletion). Controls were injected with 12 μM AllStars Negative Control siRNA or antisense LNA negative control and all embryos were co-injected with 200 ng μl^−1^ *Gap43-*GFP mRNA as an injection control. The embryos were fixed at indicated time points and assessed by immunofluorescence or subjected to RT-qPCR. See Table S1 for sequences.

#### Transient transfection

Transient transfection was carried out using either Lipofectamine RNAiMax or HiPerFect for siRNA delivery (at final concentration of 25 nM for siRNA and 15nM for stealth siRNA and ASO). All transfections were performed according to the manufacturers’ instructions and analyzed after 48h. Plasmids: the CARM1 encoding plasmid was previously described (Torres-Padilla et al., 2007). The p54nrb cDNA was obtained from Dharmacon and cloned into the pRN3p vector via BamHI/XbaI digestion and ligation. *In vitro* transcription was undertaken on linearized cDNA using the mMessage mMachine T3 kit according to the manufacturer’s instructions.

#### Immunofluorescence staining

After removal of the zona pellucida with acidic Tyrode’s solution, mouse embryos were either fixed in 3% PFA for 40 min at RT, followed by permeabilization in 0.5% Triton X-100 for 20 min or fixed in ice-cold 100% methanol for 20 min at −20°C.

Embryos were blocked overnight at 4°C in 3% BSA in PBS-T (PBS containing 0.1% Tween) and incubated with primary antibodies in blocking solution overnight at 4°C. The embryos were then washed twice in PBS-T and incubated with secondary antibodies (Alexa Fluor conjugated; 1:400 in 1.5% BSA) for 2 h before final washes in PBS-T and imaging in drops of PBS on glass-bottomed dishes, covered by paraffin oil. Blastomeres of 4-cell stage embryo were separated before imaging.

#### Proximity ligation assay

The proximity ligation assay (PLA) was performed using Duolink assay reagents. The stages prior to recognition with PLA probes were performed as for the immunofluorescence staining. Recognition with PLA probes was performed using PLA probes diluted in 3% BSA in 1x PBS for 1h at 37°C. The PLA-probes combinations were selected corresponding to the antibodies used depending on the host species of the antibodies used. Ligation of the short oligonucleotide sequences attached to the PLA probes was performed for 30 min at 37°C followed by amplification of the circular DNA formed after the ligation step for 3 h at 37°C in the dark. After the PLA procedure and a series of washes, embryos were counterstained with DAPI and imaged in drops of PBS on glass-bottomed dishes, covered by paraffin oil.

#### Western blot analysis

Cells were extracted in ice-cold lysis buffer (50 mM HEPES pH 7.5, 150 mM NaCl, 1 mM EGTA, 1 mM EDTA, 1% Triton X-100, 10% glycerol) supplemented with 5 μg/ml DNase and protease and phosphatase inhibitor cocktails. Protein concentration was measured with the BCA Protein Assay Kit and samples of 25 μg total proteins were subjected to SDS-PAGE. Resolved proteins were transferred to a nitrocellulose membrane, which was blocked with 5% BSA, and then incubated overnight at 4°C with the specific primary antibodies diluted in a blocking solution. The membrane was washed three times in PBS-T and incubated with secondary antibodies for 1 hour before final washes in PBS-T and detection with enhanced chemiluminescence (ECL).

#### qRT-PCR

Total RNA was extracted from embryos at different stages using the Arcturus PicoPure RNA isolation kit and qRT-PCR was carried out using a Power SYBR Green RNA-to-CT 1-Step Kit and a StepOne Plus Real-time PCR machine (Applied Biosystems). Relative levels of transcript expression were calculated using the ddCT method with Gapdh or Histone H3 as endogenous controls. The following primers were used: see Table S1.

#### RNA-FISH

After removal of the zona pellucida with acidic Tyrode’s solution, mouse embryos were incubated in PBS containing 6 mg/ml BSA for 15 min. Then, embryos were transferred on coverslips coated with Denhardt’s solution and dried for 30 min at room temperature (Probst et al., 2010). Embryos were fixed in 3% paraformaldehyde (PFA) for 12 min followed by permeabilization in RNA-FISH permeabilizing solution (0.5% Triton X-100, 10 mM Vanadyl Ribonucleoside Complex (VRC), in 1 × PBS) for 6 min on ice. After two washes in 70% EtOH for 5 min each, dehydration was performed in 80%, 95%, twice 100% EtOH, each for 5 min at RT, and the slides were dried for 5 min. The embryos were incubated in hybridization solution (50% formamide, 2 × SSC, 10% dextran sulfate, 10 mM VRC, 2 mg/ml BSA) containing 0.1 nM DIG-labeled RNA probes (Exiqon) at 37°C overnight. After three washes for 5 min each in a washing solution comprising 50% formamide: 50% 2 × SSC at 42°C and four washes for 5 min each in PBT (1% Tween-20, in 1 × PBS), the embryos were blocked in 10% sheep serum, 0.05% BSA, in 1 × PBS for 1 h at room temperature followed by incubation in antibody hybridization solution (2% sheep serum, 0.05% BSA, anti-DIG antibody (1:200), in 1 × PBS) for 2–3 h at room temperature. After four washes for 5 min each in PBT, embryos were stained with DAPI (10 μg/ml in PBS) for 7 min. Then, embryos were mounted on glass slides after three washes.

### Quantification and Statistical Analysis

#### Microscope analyses and image processing

Multi-channel images were acquired for multiple sections using a Leica SP5 or Leica SP8 confocal microscope (Leica Microsystems). A Plan-Apochromat lens with magnification of 63X, NA = 1.518, and a pixel size of 0,27 mm in the x- and y-direction and 1 mm in the z-direction were used. The gain and offset were adjusted to prevent over-saturation or under-saturation of an image. Laser power and detector gain were maintained constant to quantitatively compare different experimental conditions within a single experiment. The dimension of each 2D slice was 1024x1024 pixels and the gray level dynamic range of each dataset is 8 bits per pixel. For the quantification of the number of speckles and intensity a sum of slices per each image was used. In the pre-processing step the cell nucleus segmentation was carried out using the DAPI channel. We applied conventional techniques for nucleus segmentation (ImageJ software). After completing nucleus segmentation in the DAPI channel, a nucleus mask was applied to indicate which pixels belonged to nucleus and which pixels were background.

##### SPOT DETECTION

The speckle channels include some noise due to incidental and nonspecific staining. Therefore, we applied pre-processing steps through ImageJ software to suppress background noise and smooth the regions within the spots without affecting their edges. To remove the noise and smooth the foreground regions we applied anisotropic diffusion smoothing filter (Bolte and Cordelières, 2006; object counter plugin for NIH ImageJ, 2011). Subsequently we used a spot detector plugin available by an Icy software (http://icy.bioimageanalysis.org). We used the UnDecimated Wavelet Transform detector to detect spots. We indicated spots brighter than the background to be detected and specified for the detection the size of the spot between 4 to 6 pixels diameter. In those images a Wavelet Adaptive Threshold (WAT) was computed what allowed removal of the background and the remaining noise. We set a Threshold scale for 80 value (which is below the 100 value of the original WAT) and accepted a size of a spot minimum larger than the largest debris particle. The accuracy of the automated spot detection was checked manually with the first 35 nuclei.

##### TIME-LAPSE

Following injection with siRNAs, embryos were observed in a humidified chamber with 21% O_2_ and 5% CO_2_ on a spinning disc (3i) at intervals of 30 min with a z step of 5 μm. Image processing and analysis were performed with Fiji and Icy software. Image assembly was done in Illustrator CS5.

Plots and statistics were generated in GraphPad Prism, version 7.0a. Quantitative data are presented as mean ± standard error of the mean (s.e.m.) and were analyzed by using two-tailed Student’s t-test (Mann Whitney test) or ANOVA test. The p values relative to controls were marked with asterisks on the charts. For all microscopy measurements, the exact value of the number of cells used (*n*) and precision measurements used (s.e.m.) is reported in the corresponding figure legends.

## Supplemental Information

Table S1. Sequences of qRT-PCR Primers, siRNAs, Stealth siRNAs, and ASOs, Related to STAR Methods and Key Resources Table
