# Supplementary material for: CARM1 and Paraspeckles Regulate Pre-implantation Mouse Embryo Development
Source: Cell. 2018 Dec 13;175(7):1902–1916.e13. doi: 10.1016/j.cell.2018.11.027 (PMC6292842; doi:10.1016/j.cell.2018.11.027)
Supplement: Table S1. Sequences of qRT-PCR Primers, siRNAs, Stealth siRNAs, and ASOs, Related to STAR Methods and Key Resources Table [file mmc1.pdf]

Cell, Volume *175*

Supplemental Information

CARM1 and Paraspeckles Regulate Pre-implantation Mouse Embryo Development

Anna Hupalowska, Agnieszka Jedrusik, Meng Zhu, Mark T. Bedford, David M. Glover, and Magdalena Zernicka-Goetz

qRT-PCR primers		
**Name**	**Forward**	**Reverse**
Carm1	TGACATCAGTATTGTGGCACAG	CTGAGGAGCCTAAGGGAATCA
Cdx2	CAAGGACGTGAGCATGTATCC	GTAACCACCGTAGTCCGGGTA
Gapdh	TGGATTTGGACGCATTGGTC	TTTGCACTGGTACGTGTTGAT
Histone H2B	TCCGCGTTTCCGTAGTACAAC	GGGGCTGGAGCTGATTTGG
Nanog	TCTTCCTGGTCCCCACAGTTT	GCAAGAATAGTTCTCGGGATGAA
Neat1	GCTCTGGGACCTTCGTGACTCT	CTGCCTTGGCTTGGAAATGTAA
Oct3/4	CACCATCTGTCGCTTCGAGG	AGGGTCTCCGATTTGCATATCT
p54nrb	GCCAGAATGAAGGCTTGACTAT	TATCAGGGGGAAGATTGCCCA
Pspc1	CCGCCTGGAATCGAGAACAC	CTTGACCGTTAGGGCTGCT
Sox2	GCGGAGTGGAAACTTTTGTCC	CGGGAAGCGTGTACTTATCCTT

Small interfering RNA (siRNA), stealth siRNA, antisense oligonucleotides (ASO)		
**Name**	**Sequence**	**Source**
p54 siRNA	AGGCTTGACTATTGACCTGAA	Qiagen
p54 siRNA	TTGGCTTTATTCGCTTGGAAA	Qiagen
p54 siRNA	TTCCCTGCTTGTACTACTCTA	Qiagen
CARM1 stealth siRNA	CGGACTTCAAGGACAAGAT	Invitrogen
Neat1_1 ASO	TAGATTAAGACGAGGC	Exiqon
Neat1_2 ASO	AAGATGCAGCAGTCGA	Exiqon

Table S1. Oligonucleotides. Sequences of qRT-PCR primers, siRNAs, stealth siRNAs and antisense oligonucleotides (ASOs). *Related to* ***Figure STAR Methods*** *(KEY RESOURCES TABLE).*
